# Developmental evidence for parental conflict in driving *Mimulus* species barriers

**DOI:** 10.1111/nph.18438

**Published:** 2022-09-17

**Authors:** Gabrielle D. Sandstedt, Andrea L. Sweigart

**Affiliations:** ^1^ Department of Genetics University of Georgia Athens GA 30602 USA

**Keywords:** chalazal, endosperm, hybrid seed inviability, *Mimulus*, parental conflict, speciation

## Abstract

- The endosperm, a tissue that nourishes the embryo in the seeds of flowering plants, is often disrupted in inviable hybrid seeds of closely related species. A key question is whether parental conflict is a major driver of this common form of reproductive isolation.
- Here, we performed reciprocal crosses between pairs of three monkeyflower species (*Mimulus caespitosa*, *Mimulus tilingii*, and *Mimulus guttatus*). The severity of hybrid seed inviability varies among these crosses, which we inferred to be due to species divergence in effective ploidy. By performing a time series experiment of seed development, we discovered parent‐of‐origin phenotypes that provide strong evidence for parental conflict in shaping endosperm evolution.
- We found that the chalazal haustorium, a tissue within the endosperm that is found at the maternal–filial boundary, shows pronounced differences between reciprocal hybrid seeds formed from *Mimulus* species that differ in effective ploidy. These parent‐of‐origin effects suggest that the chalazal haustorium might act as a mediator of parental conflict, potentially by controlling sucrose movement from the maternal parent into the endosperm.
- Our study suggests that parental conflict in the endosperm may function as a driver of speciation by targeting regions and developmental stages critical for resource allocation and thus proper seed development.

## Introduction

Identifying the evolutionary drivers of reproductive isolation is critical for understanding the origin of species. This task has been a challenge for intrinsic postzygotic isolation, which arises when hybrids inherit novel combinations of incompatible alleles that cause inviability or sterility (Dobzhansky, 1937; Muller, 1942). Because these incompatible combinations occur uniquely in hybrids and are independent of the environment, there are usually few clues as to why the causal alleles initially increase in frequency and fix within species. In flowering plants, hybrid seed inviability is a common form of postzygotic isolation in which crosses between closely related species produce only flattened, shriveled seeds that fail to germinate (Rebernig *et al*., 2015; Oneal *et al*., 2016; Lafon‐Placette *et al*., 2017; Roth *et al*., 2018a; Coughlan *et al*., 2020; İltaş *et al*., 2021). Almost invariably, this inviable seed phenotype involves defects in the endosperm (Lafon‐Placette & Köhler, 2016), a nutritive tissue that surrounds and feeds the developing embryo. The endosperm is one of two products formed through double fertilization, a key reproductive feature of flowering plants. During this process, one of the haploid pollen sperm cells fuses with the haploid egg cell to form a diploid zygote, while the other fuses with the homodiploid central cell to form a triploid endosperm with a relative contribution of two maternal to one paternal (2m : 1p) genomes (Berger, 2003; Berger *et al*., 2008). Given its major role in postzygotic isolation, discovering how the endosperm evolves within and between closely related lineages holds great promise for probing the evolutionary mechanisms of plant speciation.

The first hints that endosperm evolution might drive reproductive barriers came from early crossing studies that showed high rates of seed failure between plants of different ploidies. Many of these studies also reported pronounced reciprocal differences in seed growth and development (Håkansson, 1952; Woodell & Valentine, 1961; Nishiyama & Inomata, 1966). In general, they found that crosses with ‘maternal excess’ – that is, crosses with the higher ploidy plant as the maternal parent – produce smaller seeds than intraploidy crosses, and that the seeds are sometimes inviable. By contrast, ‘paternal excess’ crosses – those with the higher ploidy plant as the pollen donor – generally produce larger seeds, which often abort (Scott *et al*., 1998; Pennington *et al*., 2008; Lu *et al*., 2012). These observations led to the hypothesis that seed failure is caused by a deviation from the usual dosage of 2m : 1p genomes in the triploid endosperm (Johnston *et al*., 1980; Lin, 1984; Haig & Westoby, 1989). However, because these same parent‐of‐origin effects were also discovered in interspecific crosses of the same ploidy (Cooper & Brink, 1942; Stephens, 1949; Nishiyama & Yabuno, 1978), it became clear that disruptions to the 2m : 1p ratio can also arise through allelic divergence. Thus, cross compatibility was said to be a function of ‘effective’ ploidy, rather than of absolute genome number (Johnston *et al*., 1980). In this conceptualization, plant species with higher effective ploidies have presumably accumulated genetic variation that mimics the maternal‐ and paternal‐excess effects of higher ploidy plants. Drawing on many of these same classic crossing studies, Haig & Westoby (1991) recognized that this genetic variation must affect functions specific to maternal and paternal genomes and proposed genomic imprinting – parent‐specific gene expression – as the underlying mechanism. Indeed, they argued that reciprocal differences in hybrid seed phenotypes between species diverged in effective ploidy are caused by incompatibilities that disrupt imprinted gene regulation.

In addition to offering a molecular mechanism for parent‐of‐origin effects and hybrid seed inviability, Haig & Westoby (1991) proposed the idea that parental conflict is the evolutionary driver of these phenotypes. Like the mammalian placenta, the angiosperm endosperm plays a critical role in the acquisition and transfer of nutrients to the embryo (Brink & Cooper, 1947). In plant species that receive pollen from more than one donor, the endosperm is predicted to operate as a venue for parental conflict, with maternal and paternal genomes evolving different levels of ‘preferred’ resource acquisition due to their unequal relatedness to offspring (Hamilton, 1964; Haig & Westoby, 1989; Brandvain & Haig, 2005). In a maternal (seed) parent, natural selection should favor gene expression in the endosperm that equalizes nutrient acquisition among all seeds, whereas in a paternal parent (pollen donor), selection should favor gene expression that maximizes resource acquisition in its own offspring at the expense of unrelated seeds (Haig & Westoby, 1989). At a mechanistic level, this scenario is thought to play out through epigenetic modifications during male and female gametogenesis that regulate parent‐of‐origin biased gene expression in the endosperm (i.e. genomic imprinting; Haig & Westoby, 1991; Reik & Walter, 2001; Kinoshita, 2007; Batista & Köhler, 2020). Within a population, endosperm ‘balance’ should be maintained through coevolution between loci that act to acquire resources from the seed parent and loci that moderate these acquisitive effects; however, disruptions to this balance may arise in hybrid genomes formed from species with divergent histories of parental conflict (Haig & Westoby, 1991).

According to the predictions of parental conflict theory, selection in the endosperm should target developmental timepoints or functions that are most important for nutrient uptake (Queller, 1983; Haig & Westoby, 1989). Most of what is known about the developmental phenotypes associated with hybrid seed inviability comes from crosses in *Arabidopsis* and other systems with nuclear‐type endosperms (so called because the early endosperm forms a syncytium; Floyd & Friedman, 2000; Bushell *et al*., 2003; Rebernig *et al*., 2015), where the timing of cellularization seems to be a major determinant of nutrient acquisition and seed size (Garcia *et al*., 2003; Luo *et al*., 2005; Kang *et al*., 2008; Hehenberger *et al*., 2012). In interploidy crosses in these systems, endosperm cellularization is often precocious when the seed parent has higher ploidy and delayed when the pollen parent has higher ploidy, resulting in smaller or larger seeds, respectively (Scott *et al*., 1998; Pennington *et al*., 2008; Lu *et al*., 2012; Morgan *et al*., 2021). These same maternal‐ and paternal‐excess effects on cellularization have also been observed in crosses between species of the same ploidy in *Arabidopsis* and *Capsella* (Rebernig *et al*., 2015; Lafon‐Placette *et al*., 2017, 2018), providing compelling evidence for parental conflict in these nuclear‐type endosperms. Parent‐of‐origin effects on endosperm development have also been seen in crosses between species with cellular‐type endosperms (so called because cell walls develop following the initial division of the primary endosperm nucleus). In two such systems, *Mimulus* and *Solanum*, maternal‐excess crosses appear to develop smaller endosperm cells that rapidly degrade, whereas paternal‐excess crosses develop fewer, larger endosperm cells and larger seeds (Roth *et al*., 2018a; Coughlan *et al*., 2020).

These recent studies have begun to build a strong case for the importance of parental conflict in species barriers, but few of them have explicitly investigated resource provisioning functions in distinct regions of the endosperm. In most angiosperms, the endosperm is not a homogeneous structure but rather differentiates into three spatially and functionally distinct domains: the micropylar domain that surrounds the embryo, the chalazal domain that occurs at the maternal–filial interface, and the central peripheral domain that makes up the largest portion of the endosperm (Brown *et al*., 2003). Of these domains, the micropylar and chalazal regions appear to be directly involved in nutrient transfer from maternal to filial structures (Baud *et al*., 2005; Morley‐Smith *et al*., 2008), which might make them particularly subject to manipulation by parental conflict.

Across the wildflower genus *Mimulus*, hybrid seed inviability has evolved repeatedly (Vickery, 1978; Garner *et al*., 2016; Oneal *et al*., 2016; Coughlan *et al*., 2020; Kinser *et al*., 2021; Sandstedt *et al*., 2021), making it an outstanding system for dissecting the developmental and evolutionary mechanisms of this common isolating barrier. *Mimulus* has a cellular‐type endosperm (Guilford & Fisk, 1952; Arekal, 1965; Oneal *et al*., 2016), and after a few rounds of cell division, three major domains form: the micropylar, chalazal, and central‐peripheral endosperm. The micropylar and chalazal regions give rise to separate haustoria that likely act as channels for nutrient transfer between the maternal plant and developing seed (Mikesell, 1990; Nguyen *et al*., 2000). The chalazal haustorium is ephemeral, composed of two cells extending from the ovule toward the micropylar domain that typically degenerates when the embryo is near a globular stage (Guilford & Fisk, 1952; Arekal, 1965; Oneal *et al*., 2016). At the opposite end of the seed, the two cells of the micropylar haustorium appear to penetrate the integuments (i.e. precursors of the seed coat) and degenerate when the embryo is nearly fully developed (Arekal, 1965). Given their invasion of neighboring tissues to funnel nutrients to the developing embryo, we might expect defects in the haustoria of hybrid seeds if *Mimulus* species have diverged in their levels of parental conflict. Such phenotypes have been noted before in chalazal structures of interploidy crosses in *Arabidopsis thaliana* (Scott *et al*., 1998), but they have not been described in a conflict scenario between species of the same ploidy.

In this study, we investigate the developmental phenotypes associated with hybrid seed inviability among three closely related, diploid *Mimulus* species with a nested pattern of relatedness: *Mimulus caespitosa* and *Mimulus tilingii* shared a common ancestor *c*. 382 kya, and *Mimulus guttatus* diverged from the other two *c*. 674 kya (Sandstedt *et al*., 2021). Populations of *M. caespitosa* and *M. tilingii* occur exclusively at high elevations and appear to be mostly allopatric, with *M. caespitosa* restricted to Washington state and *M. tilingii* mostly known from alpine areas of Oregon and California. *Mimulus guttatus* occupies a more diverse range in western North America, sometimes overlapping with populations of *M. caespitosa* and *M. tilingii* (Nesom, 2012; Coughlan *et al*., 2021). Previously, we showed that crosses between *M. caespitosa* and *M. tilingii* result in severe hybrid seed inviability – but only when *M. tilingii* is the paternal parent (crosses in the reciprocal direction produce mostly viable seeds, Sandstedt *et al*., 2021). Hybrid seed inviability is even stronger between the more distantly related *M. tilingii* and *M. guttatus*, which produce very few (< 1%) viable seeds in either direction of the cross (Vickery, 1978; Garner *et al*., 2016). Despite this apparent similarity between reciprocal crosses of *M. tilingii* and *M. guttatus*, most of the underlying genetic loci affect seed viability only through the maternal or paternal parent (Garner *et al*., 2016). These parent‐of‐origin effects on seed viability and genetic loci strongly point to a role for the endosperm, but its involvement has not yet been directly tested.

Here, we leverage this closely related trio of *Mimulus* species to investigate whether parental conflict is an important contributor to endosperm evolution and, potentially, hybrid seed inviability. First, we explore the severity of hybrid seed inviability in each of the three species pairs and determine whether the endosperm is involved. Second, we investigate divergence in effective ploidy among the three *Mimulus* species. For each species pair, we ask whether increasing the ploidy of one species can ‘balance’ the genetic contribution of the other and rescue hybrid seed inviability. We use this genome doubling approach to establish hierarchical relationships in effective ploidy among the three species and determine how it scales with genetic distance. Finally, we investigate the role of parental conflict in shaping this hierarchy and potentially driving species barriers. We perform detailed developmental analyses of pure species and hybrid seeds, asking whether developmental phenotypes linked to resource acquisition appear particularly affected by divergence in effective ploidy. Together, our results provide strong evidence for the involvement of parental conflict in shaping endosperm evolution, and potentially reproductive isolation, in this group of *Mimulus* species.

## Materials and Methods

### Generation of plant material

Here, we used one inbred line (formed from ≥ 8 generations of self‐fertilization) for each focal species (*Mimulus caespitosa* Greene, *Mimulus tilingii* Regel, and *Mimulus guttatus* DC). The same inbred lines were used in previous studies of hybrid seed inviability in *M. tilingii* and *M. guttatus* (Garner *et al*., 2016) and *M. caespitosa* (Sandstedt *et al*., 2021). The *M. caespitosa* inbred line, TWN36, originates from a high‐alpine population at 1594 m in Twin Lakes, WA. The *M. tilingii* inbred line, LVR1, is derived from a population at 2751 m in Yosemite Park, CA. The *M. guttatus* inbred line, DUN10, originates from a population in the Oregon Dunes National Recreation Area.

In this study, we considered three intraspecific crosses (C×C, T×T, and G×G; C, *M. caespitosa*; G, *M. guttatus*; T, *M. tilingii*) and six interspecific crosses (C×T, T×C, T×G, G×T, C×G, G×C; maternal parent is always listed first). To generate diploid, experimental plants, we sowed 20–30 seeds for each inbred line on damp paper towels in Petri dishes sealed with parafilm and cold‐stratified them for 7 d to disrupt seed dormancy. After cold stratification, we transferred the Petri dishes to a growth chamber with a 16 h : 8 h, 23°C : 16°C, light : dark photoperiod. We transplanted seedlings into 3.5 × 3.5 × 3.5‐inch pots with moist Fafard 4P Growing Mix (Sun Gro Horticulture, Agawam, MA, USA) and placed the pots in the same growth chamber. Once plants began flowering, we randomly crossed within and between individuals (total plants: C = 22, T = 20, G = 16). For all crosses, we emasculated the maternal plant 1–3 d before each cross to prevent contamination from self‐pollination.

To investigate species divergence in effective ploidy, we performed several interspecific, interploidy crosses: C_4*x*_×T, T×C_4*x*_; T_4*x*_×G, G×T_4*x*_; C_4*x*_×G, G×C_4*x*_ (4*x* subscript indicates tetraploid). To generate synthetic tetraploid individuals, we treated 100–200 seeds of TWN36 and LVR1 with 0.1% or 0.2% colchicine and stored them in the dark for 24 h (16 h at 23°C and 8 h at 16°C). The next day, we planted seeds onto Fafard 4P potting soil using a pipette and placed pots inside the growth chamber under typical light and temperature conditions (16 h : 8 h, 23°C : 16°C, light : dark photoperiod). Once the seeds germinated, we transplanted the seedlings into 2.5 × 2.5 × 2.5‐inch pots. After sufficient growth, we prepared samples for flow cytometry using a protocol adapted from Lu *et al*. (2017). Briefly, we extracted nuclei from one colchicine‐treated sample and an internal control (2*n Mimulus* or *A. thaliana*, Col‐0) together in a single well. To extract nuclei, we chopped 100 mg of leaf tissue (50 mg colchicine‐treated sample and 50 mg internal control) in 1 ml of a pre‐chilled lysis buffer (15 mM Tris–HCl pH 7.5, 20 mM NaCl, 80 mM KCl, 0.5 mM spermine, 5 mM 2‐ME, 0.2% TritonX‐100). We stained the nuclei with 4,6‐diamidino‐2‐phenylindole (DAPI), filtered them for debris using a 40 μm Flowmi™ cell strainer, and aliquoted them into a single well of a 96‐well polypropylene plate. We assessed the ploidy of each sample using a CytoFLEX (Beckman Coulter Life Sciences, Athens, GA, USA) flow cytometer. We calculated total DNA content using the following equation:

$$2C\;\mathrm{DNA}\;\text{content}\;\left(\mathrm{pg}\;\mathrm{DNA}\right)=\frac{\text{sample}\;G1\;\text{peak mean}}{\text{standard}\;G1\;\text{peak mean}}\times \text{standard}\;2C\;\mathrm{DNA}\;\text{content}$$

We generated three synthetic polyploids for TWN36 and six for LVR1. For each synthetic polyploid, 2C DNA content was nearly doubled compared to corresponding diploid lines (TWN36, 2C = 1.38 pg; TWN36_4*x*_, 2C = 2.69 ± 0.09 pg; LVR1, 2C = 1.26 pg; LVR1_4*x*_, 2C = 2.64 ± 0.05 pg). In some cases, we discovered that plants initially identified as tetraploid via flow cytometry were actually mixoploids. To ensure the crosses we performed were indeed interploidy, we determined the ploidy of the resulting progeny. From each interploidy cross, we planted 5–10 seeds per fruit, isolated nuclei from the resulting plants, and assessed 2C content using a flow cytometer for a few offspring as described in the previous paragraph (TWN36_4*x*_ × LVR1, 2C = 1.92 ± 0.04; LVR1 × TWN36_4*x*_, 2C = 1.88 ± 0.01 pg; LVR1_4*x*_ × DUN10, 2C = 1.95 ± 0.04 pg; DUN10 × LVR1_4*x*_, 2C = 1.81 ± 0.01 pg; TWN36_4*x*_ × DUN10, 2C = 1.90 ± 0.011 pg). We included data from interploidy crosses only when their progenies were confirmed to be triploids, or, in the case of 4*x M. caespitosa*, if we were using a confirmed stable polyploid line (i.e. self‐fertilized at least one generation with polyploidy confirmed in the progeny).

### Measuring seed size and seed viability

To measure seed size, we collected three replicate fruits per cross, with each fruit collected from a distinct plant. We imaged 50 seeds per fruit under a Zeiss Stemi 2000‐C Stereo microscope (Jena, Germany), for a total of 150 seeds per cross (except for one C×G fruit for which only 35 seeds were measured, for a total of 135 seeds). Seed area was measured using fiji (Schindelin *et al*., 2012).

Using these same fruits, as well as fruits from interploidy crosses (2–5 fruits per cross, at least two fruits per cross from a distinct plant), we assessed seed viability using two different methods. First, we performed visual assessments of mature seeds, looking for irregular phenotypes (shriveled, wrinkled, or flat) known to be highly correlated with germination rates in these *Mimulus* species and their hybrids (Garner *et al*., 2016; Sandstedt *et al*., 2021). We scored the number of seeds that appeared round and plump (i.e. fully‐developed) vs irregularly shaped (i.e. under‐developed). Second, we performed Tetrazolium assays to assess seed viability on a subset of these same seeds (*c*. 100 seeds per fruit). For fruits generated from interploidy crosses and fruits that produced < 100 seeds, we stained 32–63 seeds. We immersed the seeds in a scarification solution (83.3% water, 16.6% commercial bleach, and 0.1% Triton X‐100) and placed them on a shaker for 15 min. After scarification, we washed the seeds five times with water and incubated them with 1% Tetrazolium at 30°C. Two days later, we scored the number of seeds that stained dark red (viable) vs pink or white (inviable). As noted in the confirmation of triploids paragraph above, we also planted a subset of the seeds from interploidy crosses to assess ploidy, and germination rates generally reflected both seed viability measurements (data not shown).

### Seed viability rescues

To assess whether aberrant endosperm development contributes to seed defects in interspecific crosses, we attempted to rescue seed viability with a sucrose‐rich medium. We collected three fruits 8–12 d after pollination (DAP) from each intra‐ and interspecific cross (not including interploidy crosses), with each fruit collected from a distinct plant. Of the three fruits per cross, at least one fruit was collected 8 DAP (to maximize the chance of rescue). On average, we dissected 40 whole immature seeds per fruit (range = 25–57) and placed them on Petri dishes with Murashige & Skoog medium containing 4% sucrose. We sealed the Petri dishes with parafilm and placed them under constant light at 23°C for 14 d before scoring germination.

### Visualizing parent‐of‐origin effects during seed development

To compare trajectories of seed development, we performed intra‐ and interspecific crosses, and we collected fruits 3, 4, 5, 6, 8, and 10 DAP. For consistency, we performed crosses and collected fruits at the same time of day.

To visualize early seed development, we collected fruits 3 and 4 DAP (*n* = 1–2 fruits per DAP per cross) and prepared them for clearing with Hoyer's solution. We placed developing fruits in a nine parts ethanol alcohol (EtOH) : one part acetic acid fixative overnight. The following day, we washed the fruits twice in 90% EtOH for 30 min per wash. We dissected immature seeds directly from each fruit and placed them onto a microscope slide with 100 μL of three parts Hoyer's solution (70% chloral hydrate, 4% glycerol, 5% gum arabic): one part 10% gum arabic and sealed the slide with a glass cover slip. We stored the microscope slides containing cleared, immature seeds at 4°C overnight. The next day, we imaged the slides on a Leica DMRB microscope (Wetzlar, Germany) using the differential interference contrast (DIC) setting with the ×20 objective. For each fruit, we scored the number of developing seeds with and without an intact chalazal haustorium (15–56 seeds per fruit; 32–111 seeds per cross per DAP); only seeds with visible embryos were scored. Additionally, we imaged an average of 11 seeds per fruit (3–15 seeds per fruit, 10–27 seeds per cross per DAP) to assess size differences in the endosperm and chalazal haustorium at 3 and 4 DAP. For the interploidy T_4*x*_×G cross, we imaged on average 18 seeds per fruit (14–26 seeds per fruit, 29–40 seeds per cross per DAP). We outlined and measured the endosperm in all seeds and the chalazal haustorium, when present, using fiji (Schindelin *et al*., 2012). Because the chalazal haustorium was not present for all imaged seeds, sample sizes for its measurements were lower. We selected and measured images that represented typical seed development at each time point.

We defined the chalazal haustorium as two uninucleate cells that, together, form a continuous structure that penetrates toward the ovule hypostase cells (a group of tightly packed cells at the base of the ovule). To measure the chalazal haustorium, we began the outline near the epidermis of the seed (not including the hypostase cells) and extended it toward the micropylar region following the method described by Guilford & Fisk (1952) (see their fig. 27). In addition, when measuring the endosperm, we started the outline at the same position near the epidermis of the ovule and extended it toward the opening of the micropylar haustorium.

To visualize later seed development (after 4 DAP when the seed coat is too thick to clear with Hoyer's solution), we collected whole fruits at 5, 6, 8, and 10 DAP and stored them in a formaldehyde : alcohol : acetic acid fixative (10% : 50% : 5% + 35% water) for a minimum of 48 h. After fixation, we dehydrated the developing fruits with increasing concentrations of tert‐Butyl alcohol. Next, we washed the fruits three times for 2 h each with paraffin wax at 65°C before embedding them into a wax block. We sectioned the wax blocks containing whole fruits into ribbons using a Model 45 rotary microtome (Lipshaw Mfg Co., Detroit, MI, USA). Fruits collected at 5 and 6 DAP were sectioned into 12‐μm ribbons for better visualization of micropylar and chalazal domains, and fruits collected at 8 and 12 DAP were sectioned into 8‐μm ribbons. Next, we gently placed the ribbons into a warm (*c*. 40°C) water bath and positioned them on a microscope slide. We placed the slides on a slide warmer overnight to adhere the sections completely to the glass. In a staining series, we first used xylene as a clearing agent and performed several washes with increasing concentrations of EtOH to effectively stain the nuclei and cytoplasm (1% Safranin‐O and 0.5% Fast Green, respectively). We further washed the stained slides with EtOH and finished the series with xylene. We sealed the slides with a glass coverslip using Acrytol as the mounting medium.

We visualized slides using a Zeiss Axioskop 2 microscope with a ×10 objective. For each fruit, we imaged at least 10 seeds with a developing embryo per fruit (except for severe embryo‐lethal crosses: 10 DAP T×G, eight seeds imaged; 10 DAP C×G, one seed imaged). We imaged at least five consecutive sections of each seed through the embryo. For all seeds imaged at 5 and 6 DAP, we scored the presence of the chalazal haustorium. Additionally, we categorized embryo development at 6, 8, and 10 DAP into four different stages: before globular to globular, late‐globular to transition, early‐heart to late‐heart, and torpedo.

### Data analysis

We performed several statistical analyses to determine the effect of each cross on seed area, seed viability, germination success on sucrose, and area of the endosperm filled by the chalazal haustorium. For each seed phenotype, we used the R software package (Bates *et al*., 2007) to generate a linear model, a linear mixed model, or a generalized linear mixed model. Details of each model are described in Supporting Information Methods S1.

## Results

### A central role for the endosperm in *Mimulus* hybrid seed inviability

**Fig. 1:**
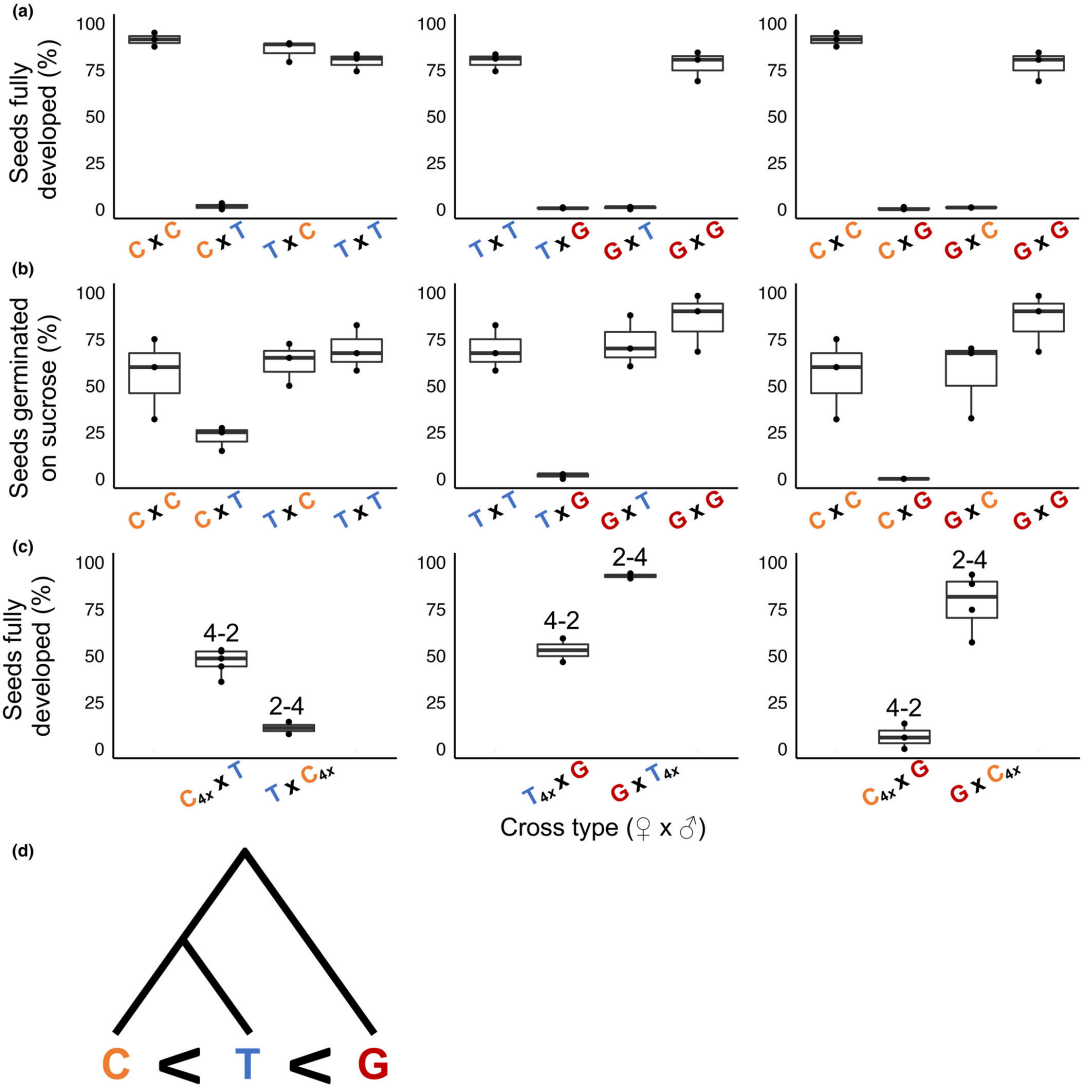
Seed viability of intra‐ and interspecific crosses among *Mimulus caespitosa* (C), *Mimulus tilingii* (T), and *Mimulus guttatus* (G). The first letter of each cross indicates the maternal species. The box and whisker plots span the distribution of all data points (shown as dots): the box contains the middle 50% of values, the whiskers represent the upper and lower 25% of values, and the horizontal line represents the median. (a) Percentage of seeds per fruit that appeared fully developed. (b) Percentage of a subset of seeds per fruit that germinated on a sucrose medium. (c) Percentage of seeds per fruit that appeared fully developed from interspecific interploidy crosses. The numbers above the box plots indicate interspecific crosses between different ploidy levels (‘4–2’, ‘2–4’) with the maternal parent's ploidy listed first. The ‘4*x*’ subscript denotes a synthetic tetraploid parent. (d) Simplified phylogenetic tree (modified from Sandstedt *et al*., 2021) with effective ploidy relationships among the three species: *M. caespitosa* is the lowest, *M. tilingii* is intermediate, and *M. guttatus* is the highest.

Hybrid seed inviability is an exceptionally strong isolating barrier in crosses between *M. guttatus*, *M. tilingii*, and *M. caespitosa* (Figs 1a, S1; Tables S1, S2). Consistent with our earlier work (Garner *et al*., 2016), *M. guttatus* and *M. tilingii* produced almost exclusively inviable F1 hybrid seeds in both directions of the cross. We found this same result in crosses between *M. guttatus* and *M. caespitosa*. On the other hand, as we have shown previously (Sandstedt *et al*., 2021), F1 hybrid seed inviability between the more closely related *M. tilingii* and *M. caespitosa* occurs in only one direction of the cross.

To investigate endosperm involvement in *Mimulus* hybrid seed failure, we attempted to rescue inviable seeds by plating them on a nutritive sucrose medium. Even when reciprocal F1 hybrid seeds appear similar in terms of morphology (i.e. flat and shriveled), supplying them with sucrose revealed clear reciprocal differences in viability (Fig. 1b; Table S3). With *M. guttatus* as the maternal parent, F1 hybrid seeds from crosses with *M. tilingii* or *M. caespitosa* germinate on sucrose at rates similar to seeds from parental crosses. By contrast, F1 hybrid seeds with *M. guttatus* as the paternal parent remain almost completely inviable even when supplied with sucrose. This result might indicate that the endosperm defect in these hybrid seeds is so severe that embryo development is irreversibly damaged. In any case, these stark reciprocal differences in F1 hybrid seed inviability – with and without sucrose – point to a central role for the endosperm in reproductive isolation between these *Mimulus* species.

### Divergence in effective ploidy among *Mimulus* species

To investigate differences in effective ploidy among this trio of *Mimulus* species, we performed a series of interploidy crosses, testing whether artificially doubling the genome content of one parent could alleviate hybrid seed inviability. Using this approach, we discovered additional support for endosperm‐based barriers and determined the rank order of effective ploidy among the three *Mimulus* species (Fig. 1c; Tables S1, S2). Consistent with *M. caespitosa* having the lowest effective ploidy, doubling its genome greatly improves hybrid seed viability in crosses with *M. tilingii* – but only when *M. caespitosa* acts as the seed parent. In the reciprocal direction, which normally produces viable seeds (Fig. 1a), 4*x M. caespitosa* pollen donors actually induce seed inviability. These results illustrate that divergence in effective ploidy can cause distinct effects through the two parental genomes: paternal excess from *M. tilingii* is severe enough to cause seed inviability, whereas maternal excess is sufficiently modest that increasing paternal dosage from *M. caespitosa* overcompensates for its effects. Along this continuum of effective ploidy, *M. guttatus* has diverged even further: 4*x M. caespitosa* restores F1 hybrid seed viability only minimally when it acts as the seed parent in crosses with this species, indicating severe paternal excess stemming from *M. guttatus*. On the other hand, maternal‐excess inviability from *M. guttatus* is not as debilitating: G×C F1 hybrid seeds are completely rescued by doubling the genome content of *M. caespitosa*. Among the three species, *M. tilingii* has an effective ploidy that is intermediate to the other two, with crosses between 4*x M. tilingii* and *M. guttatus* largely or completely restoring hybrid seed inviability. Taken together, these results demonstrate clear differences in effective ploidy: *M. guttatus* has the highest, *M. tilingii* is intermediate, and *M. caespitosa* has the lowest (Fig. 1d).

### Developmental phenotypes in *Mimulus* hybrids implicate parental conflict

To investigate whether parental conflict is the evolutionary force driving these changes in effective ploidy, our next step was to take a closer look at parent‐of‐origin seed phenotypes. As a first pass, we examined reciprocal differences in F1 hybrid seed size for each species pair, reasoning that maternal‐excess crosses might show signs of undergrowth and paternal‐excess crosses might show signs of overgrowth. Contrary to this expectation, hybrid seeds are almost always smaller than pure species seeds (except for C×T, which are the same size) and reciprocal differences are subtle or absent (Fig. S2; Table S4). However, because mature hybrid seed size depends on a multitude of developmental processes, including embryo growth and early seed abortion, it might not reflect parent‐of‐origin phenotypes operating during development.

**Fig. 2:**
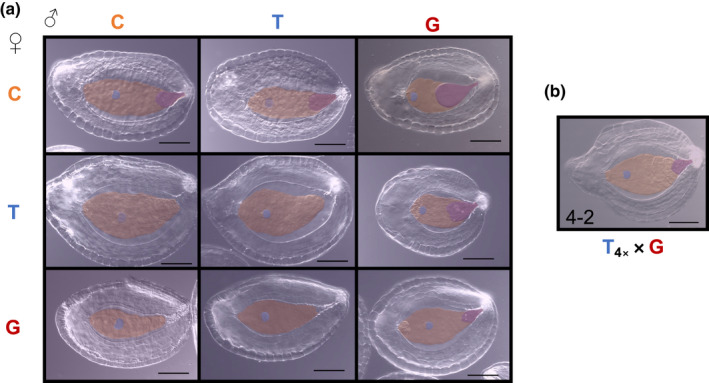
Developing seeds 4 d after pollination (DAP) in crosses among *Mimulus caespitosa* (C), *Mimulus tilingii* (T), and *Mimulus guttatus* (G). Developing seeds were cleared with Hoyer's solution. Structures were outlined and artificially shaded: blue shading represents the embryo, orange shading represents the endosperm region, and purple shading represents the chalazal haustorium. Bars, 0.1 mm. (a) Representative seeds 4 DAP of intra‐ and interspecific crosses. Maternal parent is listed along the left side, and paternal parent is listed along the top. Along the diagonal are the intraspecific crosses (C×C, T×T, and G×G), below the diagonal are maternal‐excess crosses (T×C, G×T, and G×C), and above the diagonal are paternal‐excess crosses (C×T, T×G, and C×G). (b) Representative seed of interploidy cross at 4 DAP. In the bottom left corner, ‘4–2’ indicates that the cross was between two ploidy levels, with the tetraploid maternal parent ploidy listed first. The ‘4*x*’ subscript in T_4*x*_×G further indicates that the maternal *M. tilingii* parent is a synthetic tetraploid.

**Fig. 3:**
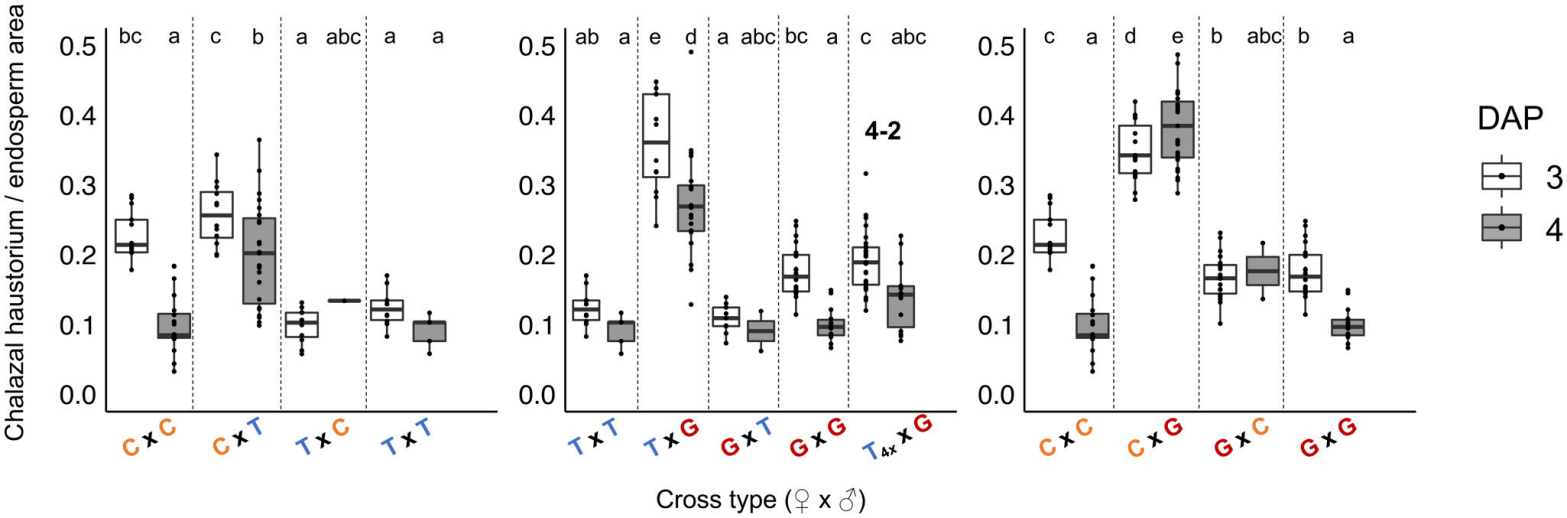
Proportion of endosperm filled by a chalazal haustorium at 3 and 4 d after pollination (DAP) in intra‐ and interspecific crosses among *Mimulus caespitosa* (C), *Mimulus tilingii* (T), and *Mimulus guttatus* (G). The first letter of each cross indicates the maternal species. The ‘4*x*’ subscript indicates a synthetic tetraploid parent. ‘4–2’ indicates that the cross was performed between two ploidy levels – tetraploid maternal parent and diploid paternal parent. The box and whisker plots span the distribution of all data points (shown as dots): the box contains the middle 50% of values, the whiskers represent the upper and lower 25% of values, with outliers falling outside of the whiskers, and the horizontal line represents the median. The white boxes represent 3 DAP and the gray boxes represent 4 DAP. We note that at 4 DAP, the *M. tilingii* parental cross and maternal‐excess crosses (T×C, G×T, G×C) have fewer data points because the chalazal haustorium was almost always absent in these crosses. Different letters above boxes indicate significant differences in least squares means among crosses (*P* < 0.05) determined using the *post hoc* Tukey method. Analyses were performed separately, comparing reciprocal interspecific and corresponding intraspecific crosses, except for crosses between *M. tilingii* and *M. guttatus*, which also include comparisons with the T_4*x*_×G cross.

**Fig. 4:**
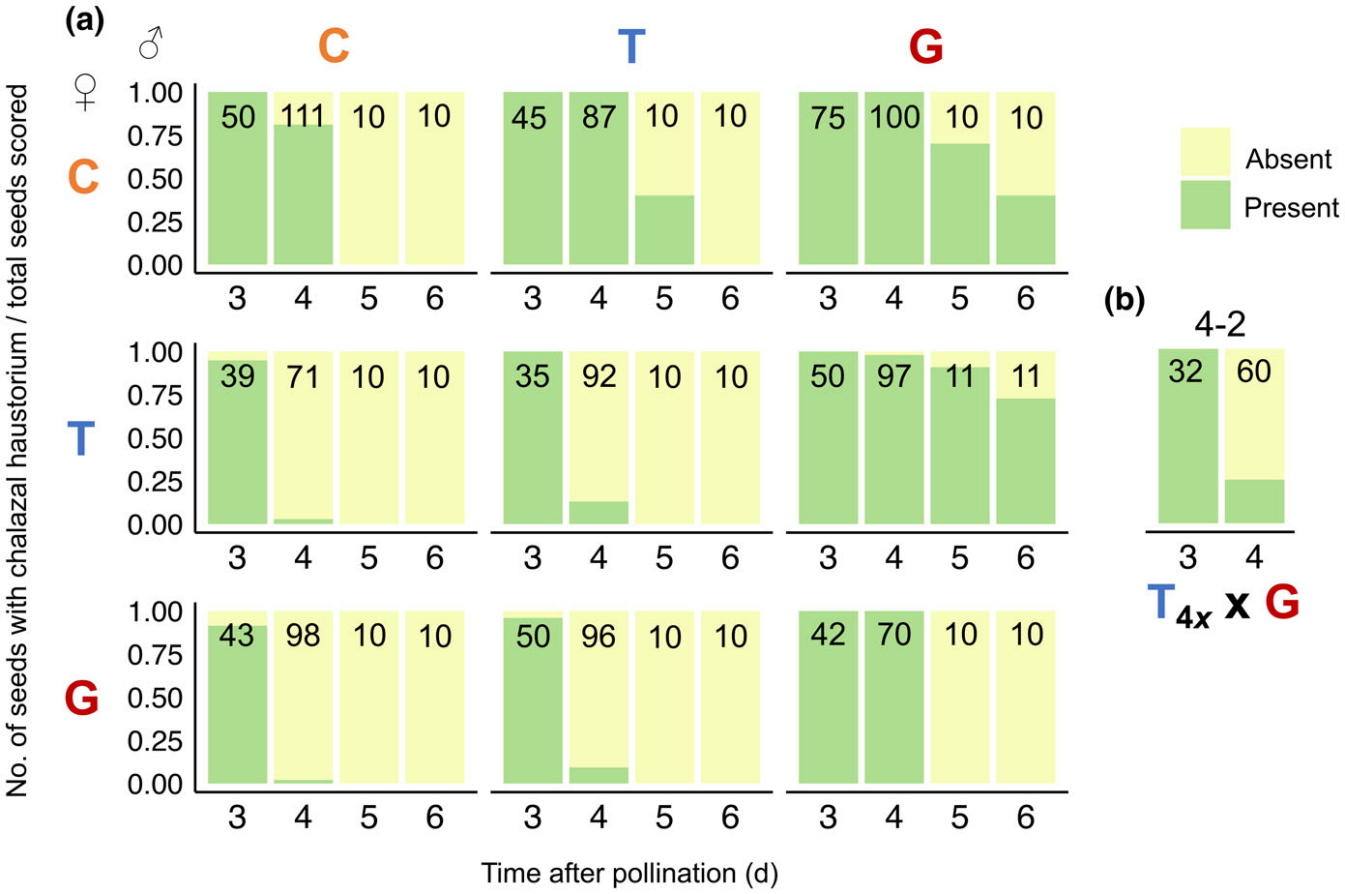
Proportion of developing seeds with a chalazal haustorium 3, 4, 5, and 6 d after pollination (DAP) from intra‐ and interspecific crosses among *Mimulus caespitosa* (C), *Mimulus tilingii* (T), and *Mimulus guttatus* (G). Numbers in bars represent the total number of developing seeds scored for a chalazal haustorium, with a subset of seeds dissected from 1 to 2 fruits per cross per DAP. Seeds were only scored and imaged if they contained a visible embryo. The green color represents the proportion of seeds with a chalazal haustorium, and the yellow color represents the proportion of seeds without a chalazal haustorium. At days 3 and 4, chalazal haustorium presence was scored after dissecting developing seeds from whole ovules and clearing them with Hoyer's solution. At days 5 and 6, chalazal haustorium presence was scored on a subset of seeds from whole‐fruit histological sections. (a) Along the diagonal are the intraspecific crosses (C×C, T×T, and G×G), below the diagonal are maternal‐excess crosses (T×C, G×T, and G×C; maternal parent always listed first), and above the diagonal are paternal‐excess crosses (C×T, T×G, and C×G). (b) In the T_4*x*_×G cross, the ‘4*x*’ subscript denotes a synthetic tetraploid *M. tilingii* maternal parent. The ‘4–2’ above the bars further represents the cross between two ploidy levels, with a tetraploid maternal parent and diploid paternal parent.

Indeed, despite superficial similarities in seed size, we observed dramatic differences in the underlying development of all reciprocal pairs of F1 hybrid seeds. In early seed development, we observed overgrowth of the chalazal haustorium in all paternal‐excess crosses (C×T, T×G, C×G in Figs 2a, S3; Table S5). Whereas during normal seed development (i.e. in the progeny of intraspecific crosses C×C, T×T, and G×G), the chalazal haustorium decreases in size early (3–4 DAP) and degenerates completely by 5 DAP, it occupies a significantly larger proportion of the endosperm in paternal‐excess crosses and is maintained much longer (Figs 3, 4, S3). In the paternal‐excess cross between *M. caespitosa* and *M. tilingii*, the volume of endosperm devoted to the chalazal haustorium at 4 DAP is nearly twice that of viable seeds (compare C×T to C×C, T×T, and T×C, Figs 2a, 3, S3; Table S5), and chalazal structures are maintained until 6 DAP (Figs 4, S4). Developmental irregularities in chalazal haustoria are even clearer in paternal‐excess crosses involving *M. guttatus*, the species with the largest effective ploidy: in T×G and C×G F1 hybrid seeds, the proportion of the endosperm filled by the chalazal haustorium is *c*. 3–4× greater than in the seeds of reciprocal and intraspecific crosses, and haustoria persist through 6 DAP (Figs 2a, 3, 4, S4; Table S5). Remarkably, this developmental defect is almost completely rescued by increasing maternal dosage. Indeed, the volume of endosperm filled by chalazal haustoria is greatly reduced in 4 × *M. tilingii* × *M. guttatus* hybrids (Figs 2b, 3; Table S5), and haustoria are almost entirely degenerated by 4 DAP (Fig. 4). In stark contrast to these paternal‐excess crosses, in maternal‐excess crosses (T×C, G×T, G×C), the chalazal endosperm degenerates precociously (Fig. 4) and is sometimes smaller early in development (3 DAP in Fig. 3).

**Fig. 5:**
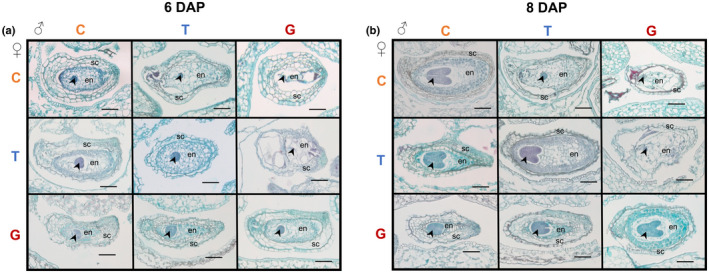
Representative whole‐fruit histological sections 6 and 8 d after pollination (DAP) from intra‐ and interspecific crosses among *Mimulus caespitosa* (C), *Mimulus tilingii* (T), and *Mimulus guttatus* (G). Maternal parent is listed along the left side, and paternal parent is listed along the top. Along the diagonal are the intraspecific crosses (C×C, T×T, and G×G), below the diagonal are maternal‐excess crosses (T×C, G×T, and G×C; maternal parent always listed first), and above the diagonal are paternal‐excess crosses (C×T, T×G, and C×G). Arrowhead, embryo; en, endosperm; sc, seed coat. Bars, 0.1 mm. (a) Six days after pollination. Intraspecific and paternal‐excess endosperms are mostly composed of large empty cells, whereas maternal‐excess crosses (especially G×T and G×C) develop endosperms that are small and composed of darkly stained, dense cells. (b) Eight days after pollination. Intraspecific endosperm cells begin to differentiate into cytoplasmically dense, starch‐filled cells along the peripheral region near the seed coat. However, in G×T and G×C crosses, the whole endosperm is composed of these dense cell types, and the endosperm remains very small and compact. Paternal‐excess endosperms appear abnormal and do not show evidence of dense cell types by 8 DAP.

**Fig. 6:**
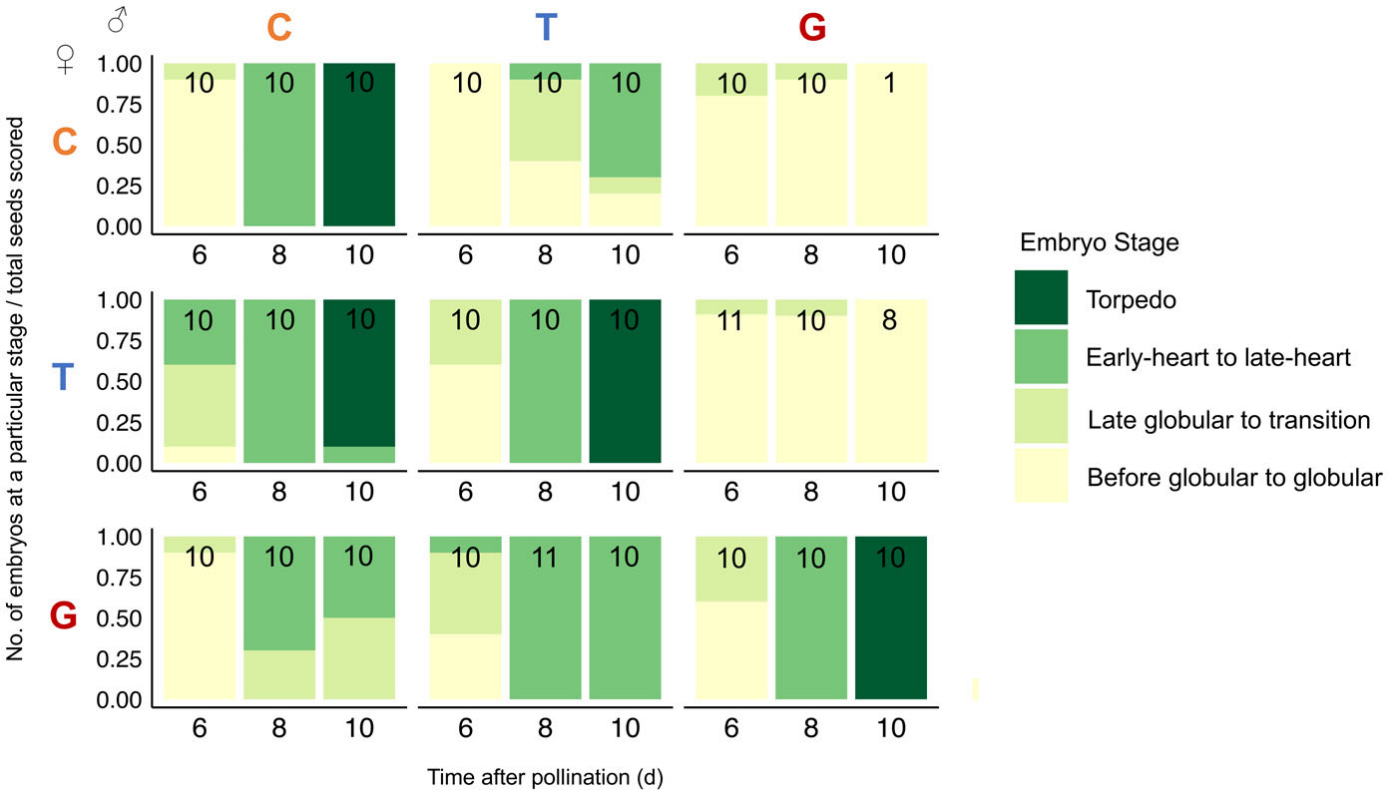
Proportion of embryos at a particular developmental stage at 6, 8, and 10 d after pollination (DAP) from intra‐ and interspecific crosses among *Mimulus caespitosa* (C), *Mimulus tilingii* (T), and *Mimulus guttatus* (G). Numbers in bars represent the total number of embryos scored per cross, where < 10 embryos suggests severe embryo lethality for a particular cross. Colors in each bar represent the stage of embryo development: yellow represents early to globular embryos, light green represents late globular to transition embryos, medium green represents early to late heart stage embryos, and dark green represents torpedo embryos. Stages of embryo development determined from whole‐fruit histological sections. Along the diagonal are the intraspecific crosses (C×C, T×T, and G×G), below the diagonal are maternal‐excess crosses (T×C, G×T, and G×C; maternal parent always listed first), and above the diagonal are paternal‐excess crosses (C×T, T×G, and C×G).

Parent‐of‐origin effects in the endosperm become even more apparent at later stages of development. At 6 DAP, the embryo of most pure species seeds is at the globular‐to‐transition‐stage and is surrounded by a cellularized endosperm with cells that appear largely empty (Figs 5a, 6, S4). By 8 DAP, the centrally located endosperm cells of these normally developing seeds appear to break down, while the peripheral endosperm lining the seed coat differentiates into cytoplasmically dense, starch‐filled cells (Figs 5b, S4: see deeply stained endosperm cells adjacent to the seed coat). However, in the seeds of maternal‐excess crosses, especially those with *M. guttatus* as the seed parent, these darkly stained endosperm cells appear earlier and are tightly packed into a smaller area (G×T and G×C at 6 DAP in Fig. 5a), and embryos fail to transition from the heart to the torpedo stage (G×T and G×C at 10 DAP in Figs 6, S4). Paternal‐excess crosses, on the other hand, produce hybrid seeds in which endosperm differentiation is either severely delayed (C×T in Figs 5, S4) or fails completely (T×G and C×G in Fig. 5). In these crosses, embryo development is also delayed (C×T in Figs 5, 6, S4) or, in the most severe cases of paternal excess (involving *M. guttatus* as the pollen parent), arrests at the globular stage (T×G and C×G in Figs 5, 6, S4).

## Discussion

Identifying the evolutionary drivers of reproductive isolation is a central goal of speciation research but remains a formidable challenge, especially for intrinsic postzygotic barriers. Our study provides some of the strongest empirical evidence to date for parental conflict as a potent force in the evolution of hybrid seed inviability. Here, we determined that three closely related *Mimulus* species differ in effective ploidy and that crosses between any species pair results in nearly complete reproductive isolation. By performing a detailed time series experiment for normal and F1 hybrid seed development, we uncovered prominent phenotypes with parent‐of‐origin effects that strongly implicate parental conflict in divergence among *M. caespitosa*, *M. tilingii*, and *M. guttatus*. This study is one of the first to detail the disruption of nutrient acquiring tissues within the endosperm from hybridizations between species of the same ploidy.

Why do we argue that the chalazal haustorium might play a special role in mediating parental conflict within a seed? In species across the angiosperm phylogeny, this specialized region of the endosperm takes on diverse forms but invariably occurs at the maternal–filial boundary, where it often projects directly into maternal tissues (Povilus & Gehring, 2022). In *Arabidopsis* and cereal crops (both with nuclear‐type endosperm development), patterns of gene expression in chalazal tissues – or in analogous endosperm transfer cells – also point to their role in nutrient transfer, with upregulation of genes involved in sugar transport and metabolism (Thiel, 2014; Zhan *et al*., 2015; Picard *et al*., 2021). In addition to this direct role in nutrient acquisition, the *Arabidopsis* chalazal endosperm appears to exert indirect effects on the process by producing the signaling protein TERMINAL FLOWER1 (TFL1), which moves to the peripheral endosperm and initiates cellularization (Zhang *et al*., 2020). Thus, mounting evidence suggests genes expressed in the chalazal region are critical in determining the amount and timing of nutrient flow into the developing embryo.

Our finding that the chalazal endosperm develops abnormally in inviable, paternal‐excess F1 hybrid *Mimulus* seeds also adds to a growing body of evidence suggesting this tissue is particularly sensitive to parental dosage and gene imprinting. Under a scenario of parental conflict in which maternally expressed genes (MEGs) and paternally expressed genes (PEGs) spar over the distribution of maternally supplied resources to the developing seeds, the chalazal endosperm should play a key role (Povilus & Gehring, 2022). In line with this prediction, gene expression of two major regulators of PEGs in *Arabidopsis* – *FIS2* and *MEA* – becomes localized in the chalazal cyst right at the point of cellularization (Luo *et al*., 2000). *FIS2* and *MEA* are themselves MEGs and members of the Polycomb Repressive Complex 2 (PRC2) complex, which act to epigenetically silence the maternal alleles of PEGs (Kinoshita *et al*., 1999; Luo *et al*., 2000; Köhler *et al*., 2005). In *fis2* mutants, endosperm cellularization fails, hexose accumulation in the central vacuole is prolonged (Hehenberger *et al*., 2012), and the chalazal endosperm is enlarged (sometimes filling *c*. 50% of the endosperm; Sørensen *et al*., 2001). This scenario of an evolutionary arms race between imprinted genes might explain why effective ploidy is positively correlated with the number and expression of PEGs in the endosperm of *Capsella* species (Lafon‐Placette *et al*., 2018), though it is important to note that this relationship is not found in wild tomato species (Roth *et al*., 2018b). Intriguingly, single nucleus RNA‐sequencing in *Arabidopsis* shows that PEG expression is specifically enriched in the chalazal endosperm (Picard *et al*., 2021). Together with our study, this evidence points toward parental conflict driving rapid changes in gene expression within the chalazal endosperm, in line with it being a particularly effective venue for manipulating the transfer of maternal resources. In further support of this idea, chalazal‐specific genes in two species of *Arabidopsis* show elevated rates of adaptive evolution compared to genes expressed in other regions of the seed (Geist *et al*., 2019). A key goal of future research will be to determine whether defects in the chalazal haustorium play a causal role in *Mimulus* hybrid seed inviability – potentially due to misregulated gene expression in that tissue – or whether it is a downstream consequence of some other dysfunctional developmental process.

In addition to the chalazal haustorium, parental conflict might manifest in other tissues in the developing seed that regulate nutrient transfer to the embryo, including the micropylar region, which transfers sucrose from the integuments to the embryo (Morley‐Smith *et al*., 2008). We found that the micropylar haustorium typically degenerates before 10 DAP in intraspecific *Mimulus* crosses but persists in some paternal‐excess crosses. For example, when *M. tilingii* acts as the seed parent and *M. guttatus* as the pollen parent, the micropylar region appears enlarged in developing hybrid seeds and is still present at 10 DAP (Fig. S4). Similar, though less severe, abnormalities also appear in C×T hybrid seeds, but a more detailed investigation of seed development in the micropylar region is needed. Intriguingly, disruptions to the micropylar region have also been reported in paternal‐excess interploidy crosses in *Galeopsis* and *Arabidopsis* (Håkansson, 1952; Scott *et al*., 1998), with micropylar haustoria vigorously invading seed integuments.

In addition to identifying the chalazal haustorium as a potential mediator of parental conflict, our study is one of only a handful to investigate divergence in effective ploidy among multiple, closely related species pairs. In this trio of *Mimulus* species, we find that effective ploidy is somewhat related to genetic distance – that is, the most closely related species pair, *M. caespitosa* and *M. tilingii*, has diverged the least in effective ploidy. However, the fact that each species has evolved to a different level of effective ploidy implies there have been lineage‐specific changes, potentially driven by differences in the strength of parental conflict. The evolution of a relatively high effective ploidy in *M. guttatus* suggests that parental conflict has either increased in this species or decreased in the lineage leading to *M. caespitosa* and *M. tilingii*. Additionally, an even lower effective ploidy in *M. caespitosa* might suggest this species has experienced a relaxation in conflict compared to *M. tilingii*. This scenario could be caused by a shift toward self‐fertilization, which theory predicts should decrease the opportunity for parental conflict (Brandvain & Haig, 2005). Indeed, mating system appears to be an important contributor to the strength of parental conflict in several systems (Rebernig *et al*., 2015; Lafon‐Placette *et al*., 2018; Raunsgard *et al*., 2018; İltaş *et al*., 2021). Although all three *Mimulus* species are hermaphroditic and self‐compatible, population genetic variation suggests they are predominantly outcrossing (Ritland & Ganders, 1987; Ritland, 1989; Ritland & Ritland, 1989; Dudash & Ritland, 1991; Willis, 1993). The strength of parental conflict within species may also depend on other factors that influence effective population size (Roth *et al*., 2019; Städler *et al*., 2021). In line with this expectation, nucleotide diversity in these three *Mimulus* species follows the same rank order as effective ploidy (Sandstedt *et al*., 2021). Even with these potentially divergent histories of conflict, disruption of the chalazal haustorium was observed in the F1 hybrid seeds of all *Mimulus* species pairs, which might suggest there have been parallel developmental changes across lineages. Going forward, identifying the genetic basis of these developmental phenotypes will be an important step toward understanding how and when parental conflict drives speciation.

## Competing interests

None declared.

## Author contributions

The research was conceived and designed by GDS and ALS, the data was collected and analyzed by GDS, and the manuscript was written by GDS and ALS. GDS and ALS contributed equally to this work.

## Supporting information


**Fig. S1** Tetrazolium assay for seed viability of intra‐, interspecific, and interploidy crosses among *Mimulus caespitosa* (C), *Mimulus tilingii* (T), and *Mimulus guttatus* (G).
**Fig. S2** Total seed area (mm^2^) of a subset of seeds per fruit from crosses within and between *M. caespitosa* (C), *M. tilingii* (T), and *M. guttatus* (G).
**Fig. S3** Developing seeds cleared with Hoyer's solution 3 and 4 d after pollination (DAP) in crosses among *M. caespitosa* (C), *M. tilingii* (T), and *M. guttatus* (G).
**Fig. S4** Histological sections of whole fruits from intra‐ and interspecific crosses among *M. caespitosa* (C), *M. tilingii* (T), and *M. guttatus* (G) at 5, 6, 8, and 10 DAP.
**Methods S1** Data analysis.
**Table S1** The effect of intra‐ and interspecific crosses among *M. caespitosa* (C), *M. tilingii* (T), and *M. guttatus* (G) on the proportion of fully‐developed seeds per fruit (scored by eye) as determined by generalized linear mixed models.
**Table S2** The effect of intra‐ and interspecific crosses among *M. caespitosa* (C), *M. tilingii* (T), and *M. guttatus* (G) on the proportion of a subset of seeds per fruit stained dark red by tetrazolium (i.e. viable seeds) as determined by generalized linear mixed models.
**Table S3** The effect of intra‐ and interspecific crosses among *M. caespitosa* (C), *M. tilingii* (T), and *M. guttatus* (G) on the proportion of a subset of immature seeds per fruit that germinated on sucrose rich media as determined by generalized linear mixed models.
**Table S4** The effect of intra‐ and interspecific crosses among *M. caespitosa* (C), *M. tilingii* (T), and *M. guttatus* (G) on seed area (mm^2^) of a subset of seeds per fruit as determined by linear mixed models.
**Table S5** The effect of intra‐ and interspecific crosses among *Mimulus caespitosa* (C), *M. tilingii* (T), and *M. guttatus* (G), days after pollination (DAP), and their interaction on the area of the endosperm filled by a chalazal haustorium (shown as a proportion) as determined by linear models.
Please note: Wiley Blackwell are not responsible for the content or functionality of any Supporting Information supplied by the authors. Any queries (other than missing material) should be directed to the *New Phytologist* Central Office.

## Data Availability

Dryad DOI for this article is doi: 10.5061/dryad.w9ghx3fs4.
