# Supplementary material for: Developmental evidence for parental conflict in driving Mimulus species barriers
Source: New Phytol. 2022 Sep 17;236(4):1545–57. doi: 10.1111/nph.18438 (PMC9826125; doi:10.1111/nph.18438)
Supplement: Supplementary file 1 — Fig. S1 Tetrazolium assay for seed viability of intra‐, interspecific, and interploidy crosses among Mimulus caespitosa (C), Mimulus tilingii (T), and Mimulus guttatus (G). Fig. S2 Total seed area (mm2) of a subset of seeds per fruit from crosses within and between M. caespitosa (C), M. tilingii (T), and M. guttatus (G). Fig. S3 Developing seeds cleared with Hoyer's solution 3 and 4 d after pollination (DAP) in crosses among M. caespitosa (C), M. tilingii (T), and M. guttatus (G). Fig. S4 Histological sections of whole fruits from intra‐ and interspecific crosses among M. caespitosa (C), M. tilingii (T), and M. guttatus (G) at 5, 6, 8, and 10 DAP. Methods S1 Data analysis. Table S1 The effect of intra‐ and interspecific crosses among M. caespitosa (C), M. tilingii (T), and M. guttatus (G) on the proportion of fully‐developed seeds per fruit (scored by eye) as determined by generalized linear mixed models. Table S2 The effect of intra‐ and interspecific crosses among M. caespitosa (C), M. tilingii (T), and M. guttatus (G) on the proportion of a subset of seeds per fruit stained dark red by tetrazolium (i.e. viable seeds) as determined by generalized linear mixed models. Table S3 The effect of intra‐ and interspecific crosses among M. caespitosa (C), M. tilingii (T), and M. guttatus (G) on the proportion of a subset of immature seeds per fruit that germinated on sucrose rich media as determined by generalized linear mixed models. Table S4 The effect of intra‐ and interspecific crosses among M. caespitosa (C), M. tilingii (T), and M. guttatus (G) on seed area (mm2) of a subset of seeds per fruit as determined by linear mixed models. Table S5 The effect of intra‐ and interspecific crosses among Mimulus caespitosa (C), M. tilingii (T), and M. guttatus (G), days after pollination (DAP), and their interaction on the area of the endosperm filled by a chalazal haustorium (shown as a proportion) as determined by linear models. Please note: Wiley Blackwell are not responsible [file NPH-236-1545-s001.pdf]

New Phytologist

***New Phytologist* Supporting Information**

**Article title:** Developmental evidence for parental conflict in driving *Mimulus* species barriers

**Authors:** Gabrielle D. Sandstedt and Andrea L. Sweigart

**Article acceptance date:** 08 August 2022

**The following Supporting Information is available for this article:**

**Methods S1** Data analysis

**Fig. S1** Tetrazolium assay for seed viability of intra-, interspecific, and interploidy crosses among *M. caespitosa* (C), *M. tilingii* (T), and *M. guttatus* (G).

**Fig. S2** Total seed area ($mm^2$) of a subset of seeds per fruit from crosses within and between *M. caespitosa* (C), *M. tilingii* (T), and *M. guttatus* (G).

**Fig. S3** Developing seeds cleared with Hoyer's solution 3 and 4 days after pollination (DAP) in crosses among *M. caespitosa* (C), *M. tilingii* (T), and *M. guttatus* (G).

**Fig. S4** Histological sections of whole fruits from intra- and interspecific crosses among *M. caespitosa* (C), *M. tilingii* (T), and *M. guttatus* (G) at 5, 6, 8, and 10 days after pollination (DAP).

**Table S1** The effect of intra- and interspecific crosses among *M. caespitosa* (C), *M. tilingii* (T), and *M. guttatus* (G) on the proportion of fully-developed seeds per fruit (scored by eye) as determined by generalized linear mixed models.

**Table S2** The effect of intra- and interspecific crosses among *M. caespitosa* (C), *M. tilingii* (T), and *M. guttatus* (G) on the proportion of a subset of seeds per fruit stained dark red by tetrazolium (*i.e.*, viable seeds) as determined by generalized linear mixed models.

**Table S3** The effect of intra- and interspecific crosses among *M. caespitosa* (C), *M. tilingii* (T), and *M. guttatus* (G) on proportion of a subset of immature seeds per fruit that germinated on sucrose rich media as determined by generalized linear mixed models.

**Table S4** The effect of intra- and interspecific crosses among *M. caespitosa* (C), *M. tilingii* (T), and *M. guttatus* (G) on seed area ($mm^2$) of a subset of seeds per fruit as determined by linear mixed models.

**Table S5** The effect of intra- and interspecific crosses among *M. caespitosa* (C), *M. tilingii* (T), and *M. guttatus* (G) on days after pollination (DAP) and their interaction on the area of the endosperm filled by a chalazal haustorium (shown as a proportion) as determined by linear models.

**Methods S1** ***Data Analysis***

We modeled the effect of cross on seed area ($mm^2$) using three separate linear mixed models, each with four comparisons including reciprocal interspecific crosses and the corresponding intraspecific crosses (CxC, CxT, TxC, TxT; TxT, TxG, GxT, GxG; and CxC, CxG, GxC, GxG). For each model, we fit a Gaussian distribution using the lmer command in the "lme4" package implemented in R (Bates *et al.*, 2007). We assigned our fixed factor as cross, random factor as individual plant, and our response variable as seed area ($mm^2$). To determine whether there was an effect of cross on the variance of seed area, we computed an ANOVA test using the anova function in the R package "car" with type III sums of squares, which applies Wald chi-square tests for mixed models. We calculated least-squares means (lsmeans) using the emmeans function in the R package "emmeans", performed pairwise comparisons between all crosses, and we used a post hoc Tukey method adjustment to determine which crosses differed significantly in seed area (Lenth & Lenth, 2018).

We determined the effect of cross on seed viability using three generalized linear mixed models (GLMMs), for both measures of seed viability (visual and tetrazolium assessment). Each GLMM compared reciprocal interspecific crosses, and their corresponding interploidy and intraspecific crosses (CxC, CxT, TxC, TxT, $C_{4x}$xT, Tx$C_{4x}$; TxT, TxG, GxT, GxG, $T_{4x}$xG, Gx$T_{4x}$; and CxC, CxG, GxC, GxG, $C_{4x}$xG, Gx$C_{4x}$). In these models, we fit GLMMs with a binomial distribution using the glmer command in the "lme4" package implemented in R (Bates *et al.*, 2007). For our response variable, we combined the number of viable seeds (fully-developed or stained dark red) and the number of inviable seeds (under-developed or unstained) into a single variable using the R function cbind. We assigned our fixed factor as cross, and the individual plant was set as a random factor. We computed ANOVAs using the anova function to determine whether cross significantly affected the variance of seed viability. Then, we calculated lsmeans and performed pairwise comparisons between all crosses. We determined which crosses differed significantly in the number of viable seeds using a post hoc Tukey method adjustment.

To model the effect of cross on germination success of seed viability rescues with sucrose media, we performed three separate GLMMs, comparing only reciprocal interspecific crosses and their corresponding intraspecific crosses (CxC, CxT, TxC, TxT; TxT, TxG, GxT, GxG; and

CxC, CxG, GxC, GxG). In these models, we fit GLMMs with a binomial distribution using the glmer command. For our response variable, we combined the number of seeds that germinated and the number of seeds that failed to germinate on a sucrose-rich medium into a single variable using the R function cbind. We assigned our fixed factor as cross, and the individual plant was set as a random factor. We computed an ANOVA to determine which crosses significantly affected variance of germination success on a sucrose-rich medium using the anova function in R. Similar to prior analyses, we estimated lsmeans, performed pairwise comparisons of lsmeans between all crosses, and determined which crosses significantly differed in the number of seeds that germinated on a sucrose-rich medium using a post hoc Tukey method.

To determine whether cross had a significant effect on area of endosperm filled by a chalazal haustorium, we performed three separate linear models for both measurements, comparing only reciprocal interspecific crosses and their corresponding intraspecific crosses—except for T-G comparisons, in which case we also included measurements of the interploidy cross (CxC, CxT, TxC, TxT; TxT, TxG, GxT, GxG, $T_{4x}$xG; and CxC, CxG, GxC, GxG). We fit linear models using the lm function in R, assigning the response variable as either chalazal haustorium/endosperm area and fixed factors as cross, DAP, and their interaction. To determine whether these fixed factors affected the variance of the response variables, we computed ANOVAs with type III sums of squares. Then, we estimated lsmeans, performed pairwise comparisons of lsmeans, and determined which crosses at 3 and 4 DAP differed in embryo area and area of the endosperm filled by the chalazal haustorium.

## References

**Bates D, Sarkar D, Bates MD, Matrix L. 2007.** The lme4 package. *R package version* **2**: 74.

**Lenth R, Lenth MR. 2018.** Package ‘lsmeans’. *The American Statistician* **34**: 216–221.

## Supporting Figures

**Fig. S1** Tetrazolium assay for seed viability of intra-, interspecific, and interploidy crosses among *M. caespitosa* (C), *M. tilingii* (T), and *M. guttatus* (G). The box and whisker plots span the distribution of data points (shown as dots): the box contains the middle 50% of values, the whiskers represent the upper and lower 25% of values, and the horizontal line represents the median. Any outliers fall outside whiskers. **(a)** Example of tetrazolium test on seeds from intra and interspecific crosses of *M. tilingii* and *M. caespitosa.* Intraspecific crosses: TxT (top left) and CxC (bottom right). Interspecific crosses, maternal parent is always listed first: CxT (bottom left), TxC (top right). Dark red seeds are scored as viable, and pink or white seeds are scored as inviable. Scale bar is 1 mm. **(b)** Percent seeds of a subset of seeds per fruit that stained dark red from intra- and interspecific crosses. **(c)** Percent seeds of a subset of seeds per fruit that stained dark red from interploidy crosses. The numbers above the boxes indicate interspecific crosses between different ("4-2", "2-4") ploidy levels with the maternal parent's ploidy listed first. "4x" subscript denotes synthetic tetraploid parent. Note that for some interploidy crosses, 5-10 fully-developed seeds were planted to test for ploidy prior to tetrazolium assay.

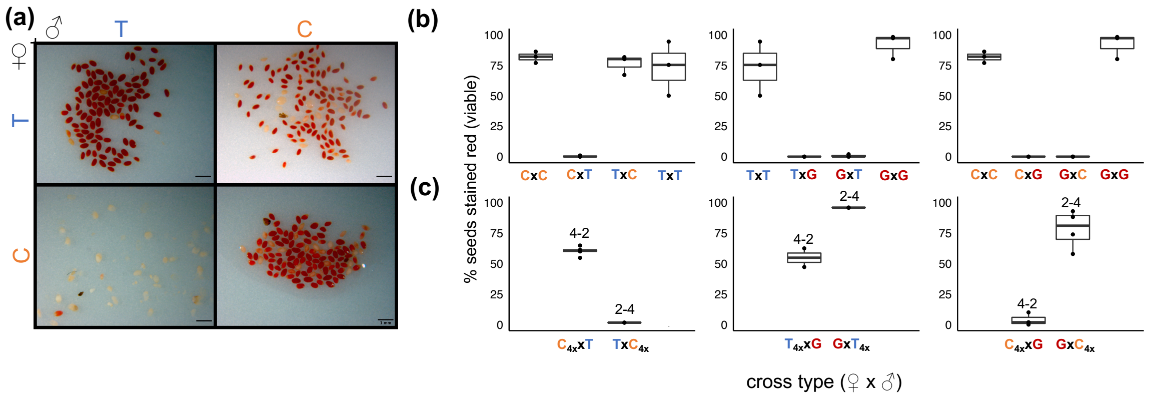

**Fig. S2** Total seed area ($mm^2$) of a subset of seeds per fruit from crosses within and between *M. caespitosa* (C), *M. tilingii* (T), and *M. guttatus* (G). Shaded violin plots show the distribution of all the data points. Data points were jittered to prevent overplotting. The first letter of each cross indicates the maternal species. Different letters indicate significant differences in least-squares means among crosses (P<0.05) determined by a post hoc Tukey method. Analyses were performed separately, only comparing reciprocal interspecific crosses and their corresponding intraspecific crosses.

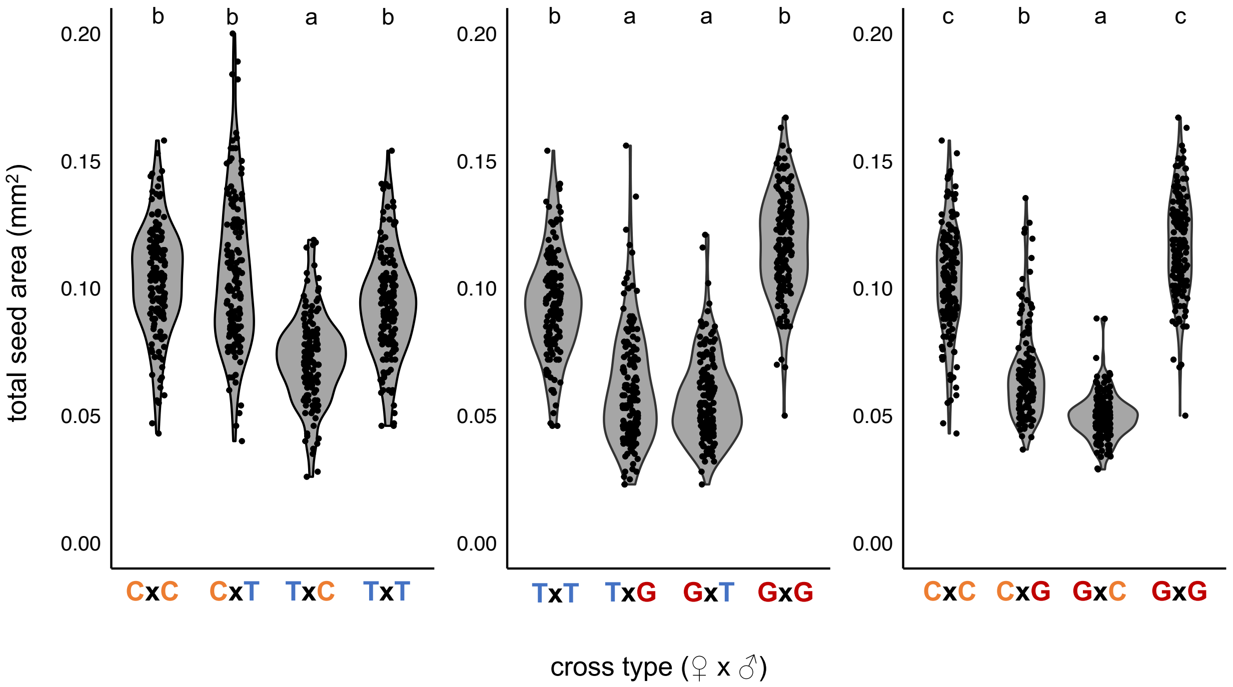

**Fig. S3** Developing seeds cleared with Hoyer's solution 3 and 4 days after pollination (DAP) in crosses among *M. caespitosa* (C), *M. tilingii* (T), and *M. guttatus* (G). In addition, we included ($T_{4x}$xG), with the 4x subscript indicating the tetraploid parent. Maternal parent is listed first in interspecific crosses. Seeds were cleared with Hoyer's solution. The first two columns include structures that were outlined and artificially shaded: blue shade represents embryo, orange shade represents endosperm region, and purple shade represents chalazal haustorium. The last two columns are the unshaded seeds from the first two columns. Scale bar is 0.1mm. At 3 DAP, chalazal and micropylar haustoria are fully established. The micropylar domain is composed of two cells (mh label points to two nuclei in micropylar region of the TxT seed) at the anterior end of the seed, and this region invades nearby seed integuments. We also sometimes observed the micropylar haustorium extending towards the chalazal domain (see GxG, GxT, and GxC). The chalazal haustorium is composed of two cells that occupy the posterior end of the seed. The chalazal haustorium extends from the maternal-filial boundary towards the anterior end of the seed. At 4 DAP, the chalazal haustorium has largely degenerated in TxT, TxC, GxT, GxC, and $T_{4x}$xG crosses, as the central endosperm proliferates. The area of the endosperm that is filled by the chalazal haustorium decreases from 3 to 4 DAP in almost all crosses, except for CxG.

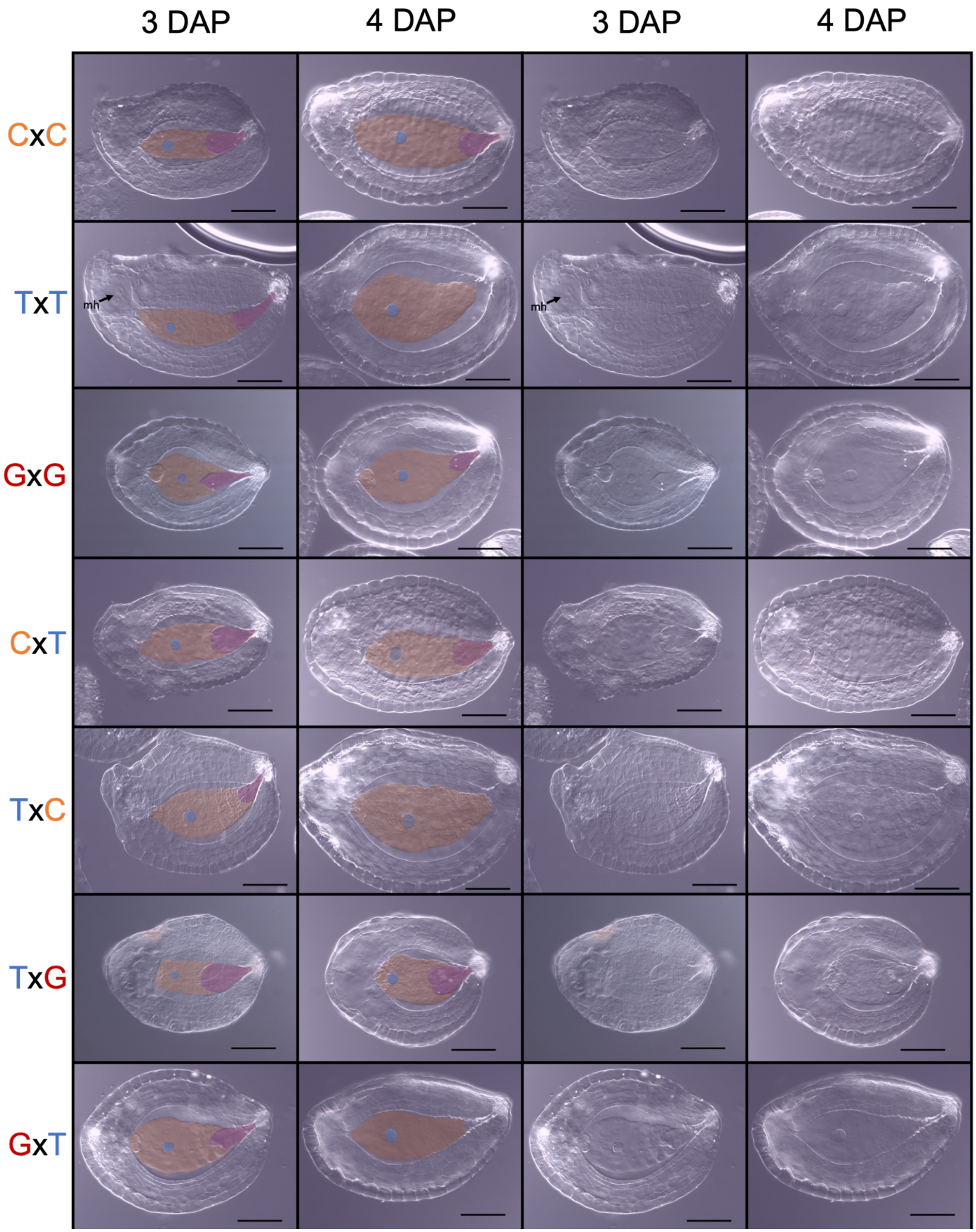
3 DAP
4 DAP
3 DAP
4 DAP
CxC
TxT
GxG
CxT
TxC
TxG
GxT
mh
mh

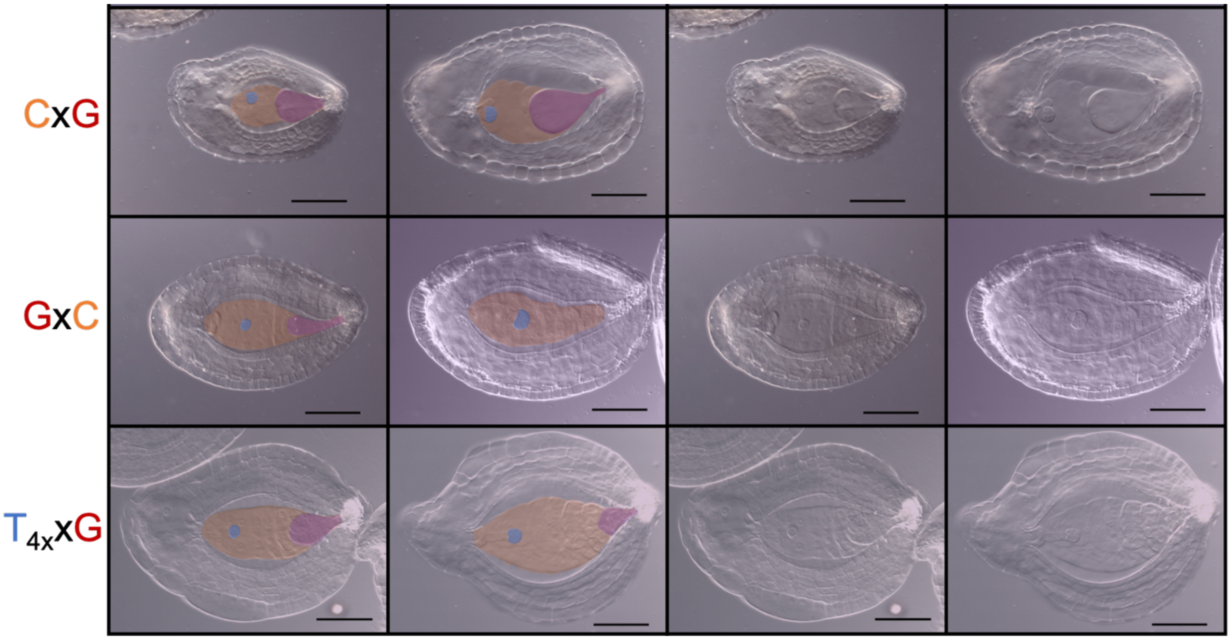

CxG
GxC
T4xxG

**Fig. S4** Histological sections of whole fruits from intra- and interspecific crosses among *M. caespitosa* (C), *M. tilingii* (T), and *M. guttatus* (G) at 5, 6, 8, and 10 days after pollination (DAP). Maternal parent is listed first in all interspecific crosses. Arrowhead = embryo, en = endosperm, sc = seed coat, ch = chalazal haustorium, mh = micropylar haustorium. Note that ch and mh are only labeled when haustoria are visible in the image. Scale bar is 0.1mm. At **5 and 6 DAP**, intraspecific crosses have reached a globular embryo stage, where the embryo is surrounded by 'empty' cells, and the chalazal haustorium has degenerated. Embryos of GxT and GxC maternal excess crosses are surrounded by dense, starch-filled cells, again with no chalazal haustorium present. In paternal-excess crosses (CxT, TxG, and CxG), embryos have not yet reached a full globular stage, the chalazal haustorium is still intact in some seeds of paternal-excess crosses at 5 DAP and in TxG and CxG at 6 DAP. We also note here that chalazal haustorium of CxT and TxG are deeply stained, likely with sugars, while the CxG haustorium is large and unstained. At **8 DAP**, intraspecific and maternal-excess crosses have reached the heart shaped embryo stage, though heart embryos of maternal-excess crosses GxT and GxC appear abnormal. While in the intraspecific crosses, the central endosperm cells begin to break down and the peripheral endosperm near the seed coat starts to differentiate into starch-filled cells, the maternal-excess crosses appear fully differentiated and the endosperm area appears reduced. In contrast, endosperm cells in paternal-excess crosses remain empty and enlarged, and embryos are underdeveloped. By **10 DAP**, all intraspecific crosses and TxC have developed torpedo shaped embryos surrounded by a few layers of dense, starch-filled cells, and micropylar haustoria are completely degenerated. The maternal-excess crosses, GxT and GxC, fail to develop torpedo shaped embryos and remain as abnormal heart shaped embryos, with little to no endosperm and no apparent micropylar haustorium. In CxT, the embryo has finally reached a heart shape, but the endosperm cells remain undifferentiated and micropylar cells are evident in some seeds. While TxG seeds are severely underdeveloped, with prominent micropylar haustorium in some seeds, the seeds of CxG crosses have already collapsed around the underdeveloped embryo.

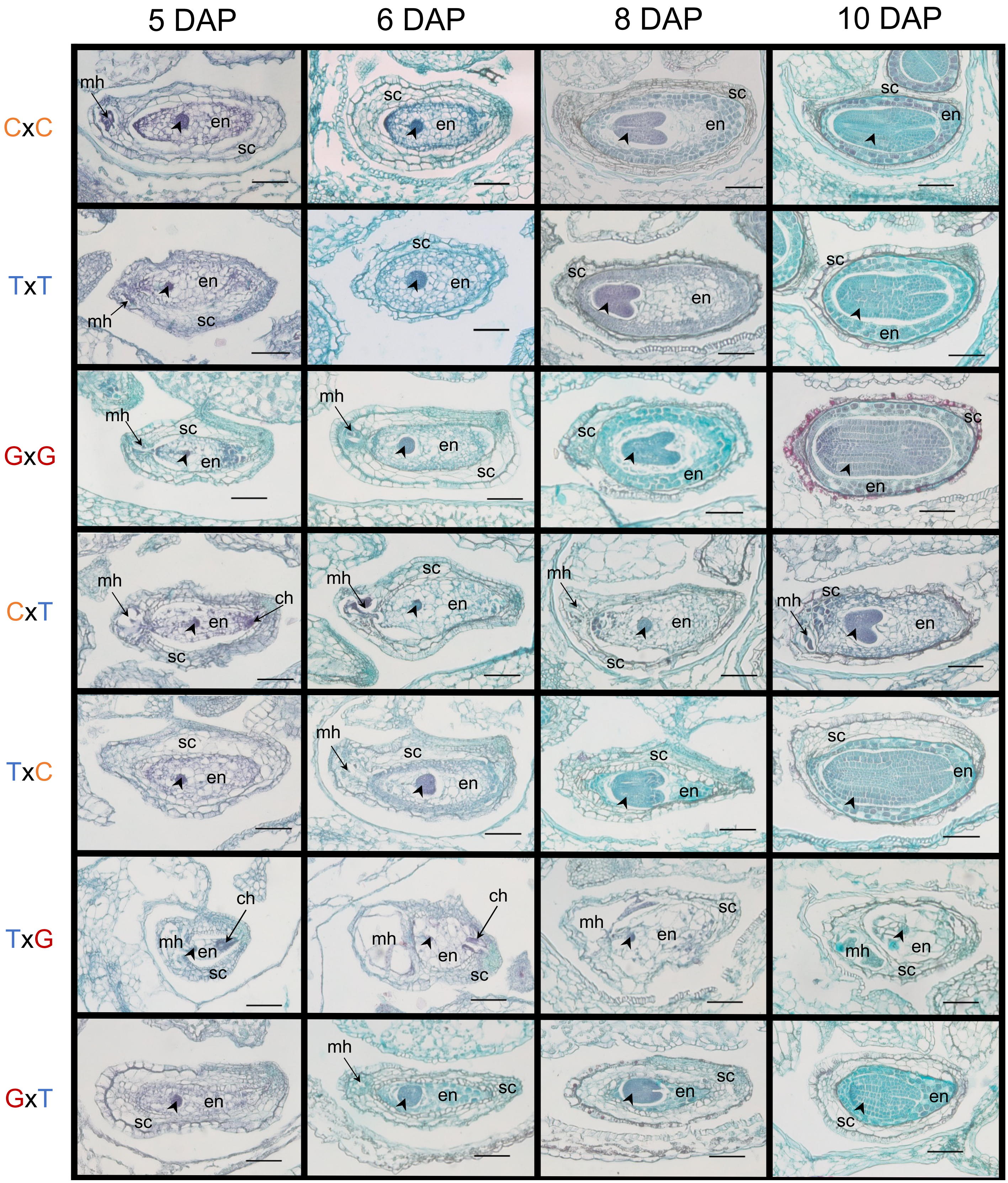
5 DAP
6 DAP
8 DAP
10 DAP
CxC
TxT
GxG
CxT
TxC
TxG
GxT
mh
en
sc
ch

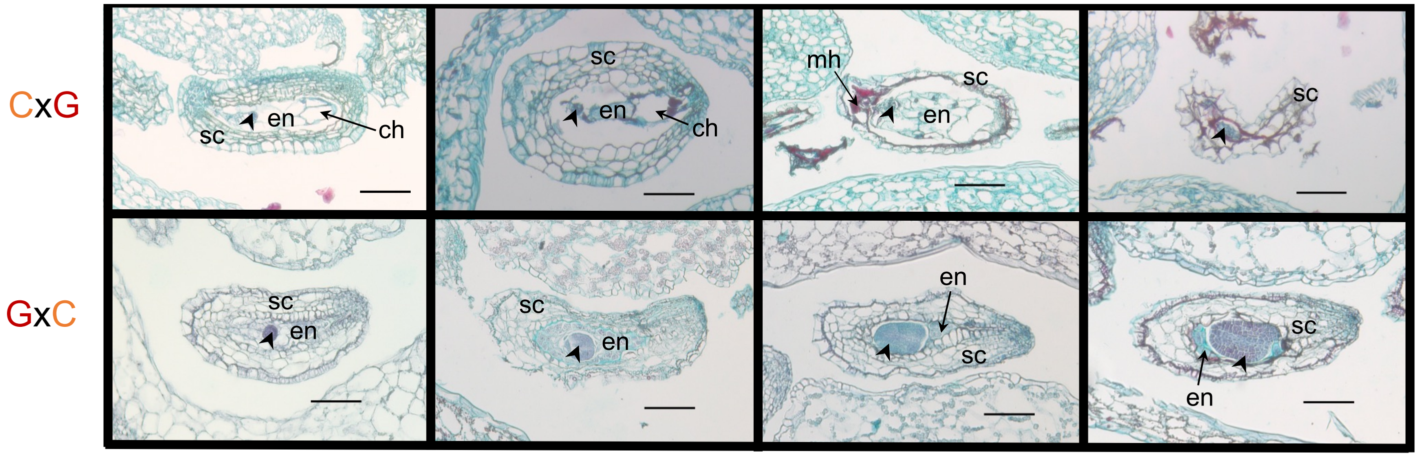

CxG
GxC
sc
en
ch
mh

## Supporting Tables

**Table S1** The effect of intra- and interspecific crosses among *M. caespitosa* (C), *M. tilingii* (T), and *M. guttatus* (G) on the proportion of fully-developed seeds per fruit (scored by eye) as determined by generalized linear mixed models. In these models, we also included interspecific, interploidy crosses (as denoted by "4-2" and "2-4", with the first number indicating the maternal parent's ploidy level). For all interspecific crosses, maternal parent is always listed first. Three separate models were performed, each with six comparisons including reciprocal interspecific crosses, interspecific interploidy crosses, and their corresponding intraspecific crosses (CxC, CxT, CxT 4-2, TxC, TxC 2-4, TxT; TxT, TxG, TxG 4-2, GxT, GxT 2-4, GxG; and CxC, CxG, CxG 4-2, GxC, GxC 2-4, GxG). On the left, output from an ANOVA, with type III sums of squares, chi-square (χ2), degrees of freedom (df), and p-values calculated using likelihood ratio tests. On the right, least-squares means (lsmeans) and standard error (SE) for each cross. Lsmeans denoted by a different letter (under "group") indicates significant differences among crosses ($P$ <0.05) determined by post-hoc Tukey method.

| ANOVA | | | | LSMEANS TUKEY-METHOD COMPARISON | | | |
|---|---|---|---|---|---|---|---|
| fixed factor | Likelihood ratio (χ2) | df | *P* | cross type | lsmeans | SE | group |
| cross | 429.08 | 5 | 2E-16 | **CxC** | 0.918 | 0.014 | e |
| | | | | **CxT** | 0.015 | 0.005 | a |
| | | | | **CxT 4-2** | 0.482 | 0.042 | c |
| | | | | **TxC** | 0.873 | 0.022 | e |
| | | | | **TxC 2-4** | 0.111 | 0.033 | b |
| | | | | **TxT** | 0.769 | 0.032 | d |
| cross | 400.72 | 5 | 2.2E-16 | **TxT** | 0.794 | 0.028 | c |
| | | | | **TxG** | 0.007 | 0.003 | a |
| | | | | **TxG 4-2** | 0.519 | 0.058 | b |
| | | | | **GxT** | 0.009 | 0.004 | a |
| | | | | **GxT 2-4** | 0.928 | 0.018 | d |
| | | | | **GxG** | 0.789 | 0.025 | c |
| cross | 296.69 | 5 | 2.2E-16 | **CxC** | 0.918 | 0.024 | c |
| | | | | **CxG** | 0.005 | 0.003 | a |
| | | | | **CxG 4-2** | 0.072 | 0.029 | b |
| | | | | **GxC** | 0.008 | 0.004 | a |
| | | | | **GxC 2-4** | 0.859 | 0.039 | c |
| | | | | **GxG** | 0.786 | 0.051 | c |

**Table S2** The effect of intra- and interspecific crosses among *M. caespitosa* (C), *M. tilingii* (T), and *M. guttatus* (G) on the proportion of a subset of seeds per fruit stained dark red by tetrazolium (*i.e.*, viable seeds) as determined by generalized linear mixed models. In these models, we also included interspecific, interploidy crosses (as denoted by "4-2" and "2-4" with the first number indicating the maternal parent's ploidy level). For all interspecific crosses, maternal parent is always listed first. Three separate models were performed, each with six comparisons including reciprocal interspecific crosses, interspecific interploidy crosses, and their corresponding intraspecific crosses (CxC, CxT, CxT 4-2, TxC, TxC 2-4, TxT; TxT, TxG, TxG 4-2, GxT, GxT 2-4, GxG; and CxC, CxG, CxG 4-2, GxC, GxC 2-4, GxG). On the left, output from an ANOVA, with type III sums of squares, chi-square ($\chi 2$), degrees of freedom (df), and p-values calculated using likelihood ratio tests. On the right, least-squares means (lsmeans) and standard error (SE) for each cross. Lsmeans denoted by a different letter (under "group") indicates significant differences among crosses ($P$ <0.05) determined by post-hoc Tukey method. Asterisks under "group" denotes insufficient variation in response variable to determine statistical differences.

| ANOVA | | | | LSMEANS TUKEY-METHOD COMPARISON | | | |
|---|---|---|---|---|---|---|---|
| fixed factor | Likelihood ratio ($\chi 2$) | df | *P* | cross types | lsmeans | SE | group |
| cross | 96.14 | 5 | 2E-16 | **CxC** | 0.824 | 0.046 | b |
| | | | | **CxT** | 0.003 | 0.003 | a |
| | | | | **CxT 4-2** | 0.611 | 0.075 | b |
| | | | | **TxC** | 0.831 | 0.040 | b |
| | | | | **TxC 2-4** | 0.014 | 0.011 | a |
| | | | | **TxT** | 0.750 | 0.052 | b |
| cross | 94.44 | 5 | 2.22E-16 | **TxT** | 0.777 | 0.083 | bc |
| | | | | **TxG** | 0.000 | 0.000 | abc* |
| | | | | **TxG 4-2** | 0.548 | 0.148 | b |
| | | | | **GxT** | 0.006 | 0.005 | a |
| | | | | **GxT 2-4** | 0.964 | 0.027 | c |
| | | | | **GxG** | 0.938 | 0.030 | c |
| cross | 57.34 | 5 | 4.30E-11 | **CxC** | 0.821 | 0.061 | b |
| | | | | **CxG** | 0.000 | 0.000 | ab* |
| | | | | **CxG 4-2** | 0.037 | 0.024 | a |
| | | | | **GxC** | 0.000 | 0.000 | ab* |
| | | | | **GxC 2-4** | 0.819 | 0.063 | b |
| | | | | **GxG** | 0.945 | 0.025 | b |

**Table S3** The effect of intra- and interspecific crosses among *M. caespitosa* (C), *M. tilingii* (T), and *M. guttatus* (G) on proportion of a subset of immature seeds per fruit that germinated on sucrose rich media as determined by generalized linear mixed models. Three separate models were performed, each with four comparisons including reciprocal interspecific crosses and their corresponding intraspecific crosses (CxC, CxT, TxC, TxT; TxT, TxG, GxT, GxG; and CxC, CxG, GxC, GxG). On the left, output from an ANOVA, with type III sums of squares, chi-square (χ2), degrees of freedom (df), and p-values calculated using likelihood ratio tests. On the right, least-squares means (lsmeans) and standard error (SE) for each cross. Lsmeans denoted by a different letter (under ”group”) indicates significant differences among crosses (*P* <0.05) determined by post-hoc Tukey method. Asterisk under “group” denotes insufficient variation in response variable to determine statistical differences.

| ANOVA | | | | LSMEANS TUKEY-METHOD COMPARISON | | | |
|---|---|---|---|---|---|---|---|
| fixed factor | Likelihood ratio (χ2) | df | p | cross type | lsmeans | SE | group |
| cross | 29.91 | 3 | 1.44E-06 | **CxC** | 0.578 | 0.071 | b |
| | | | | **CxT** | 0.221 | 0.052 | a |
| | | | | **TxC** | 0.628 | 0.066 | b |
| | | | | **TxT** | 0.698 | 0.061 | b |
| cross | 41.68 | 3 | 4.7E-09 | **TxT** | 0.688 | 0.092 | b |
| | | | | **TxG** | 0.017 | 0.014 | a |
| | | | | **GxT** | 0.754 | 0.079 | b |
| | | | | **GxG** | 0.862 | 0.054 | b |
| cross | 13.82 | 3 | 3.15E-03 | **CxC** | 0.569 | 0.124 | ab |
| | | | | **CxG** | 0.000 | 1.8e-0.7 | ab* |
| | | | | **GxC** | 0.487 | 0.118 | a |
| | | | | **GxG** | 0.894 | 0.050 | b |

**Table S4** The effect of intra- and interspecific crosses among *M. caespitosa* (C), *M. tilingii* (T), and *M. guttatus* (G) on seed area ($mm^2$) of a subset of seeds per fruit as determined by linear mixed models. For all interspecific crosses, maternal parent is always listed first. Three separate models were performed, each with four comparisons including reciprocal interspecific and their corresponding intraspecific crosses (CxC, CxT, TxC, TxT; TxT, TxG, GxT, GxG; and CxC, CxG, GxC, GxG). On the left, output from an ANOVA, with type III sums of squares, chi-square (χ2), degrees of freedom (df), and p-values calculated using likelihood ratio tests. On the right, least-squares means (lsmeans) and standard error (SE) for each cross. Lsmeans denoted by a different letter (under ”group”) indicates significant differences among crosses ($P$ <0.05) determined by post-hoc Tukey method.

| ANOVA | | | | LSMEANS TUKEY-METHOD COMPARISON | | | |
|---|---|---|---|---|---|---|---|
| fixed factor | Likelihood ratio (χ2) | df | p | cross types | lsmeans | SE | group |
| cross | 27.79 | 3 | 4.02E-06 | **CxC** | 0.105 | 0.006 | b |
| | | | | **CxT** | 0.109 | 0.006 | b |
| | | | | **TxC** | 0.076 | 0.006 | a |
| | | | | **TxT** | 0.094 | 0.006 | b |
| cross | 464.53 | 3 | 2.22E-16 | **TxT** | 0.094 | 0.005 | b |
| | | | | **TxG** | 0.061 | 0.005 | a |
| | | | | **GxT** | 0.059 | 0.004 | a |
| | | | | **GxG** | 0.114 | 0.004 | b |
| cross | 176.16 | 3 | 2.22E-16 | **CxC** | 0.103 | 0.004 | c |
| | | | | **CxG** | 0.068 | 0.004 | b |
| | | | | **GxC** | 0.050 | 0.004 | a |
| | | | | **GxG** | 0.117 | 0.004 | c |

**Table S5** The effect of intra- and interspecific crosses among *M. caespitosa* (C), *M. tilingii* (T), and *M. guttatus* (G) on days after pollination (DAP) and their interaction on the area of the endosperm filled by a chalazal haustorium (shown as a proportion) as determined by linear models. For all interspecific crosses, maternal parent is always listed first. Three separate models were performed, two models had eight comparisons, including reciprocal interspecific crosses and their corresponding intraspecific crosses (3 and 4 DAP: CxC, CxT, TxC, TxT; CxC, CxG, GxC, GxG). One model had 10 comparisons, including reciprocal interspecific crosses, one interploidy cross with a tetraploid maternal and diploid paternal parent, and their corresponding intraspecific crosses (3 and 4 DAP: TxT; TxT, TxG, TxG 4-2, GxT, GxG). On the left, output from an ANOVA with type III sums of squares, sums of squares (SS), degrees of freedom (df), and p-values. On the right, least-squares means (lsmeans) and standard error (SE) for each cross. Lsmeans denoted by a different letter (under "group") indicates significant differences among crosses ($P$ <0.05) determined by post-hoc Tukey method.

| ANOVA | | | | | LSMEANS TUKEY-METHOD COMPARISON | | | | |
|---|---|---|---|---|---|---|---|---|---|
| fixed factor | sum of squares (SS) | df | F-value | p | cross type | DAP | lsmeans | SE | group |
| | | | | | **CxC** | **3 DAP** | 0.228 | 0.012 | bc |
| | | | | | **CxC** | **4 DAP** | 0.098 | 0.012 | a |
| cross | 0.22 | 3 | 30.43 | 2.05E-13 | **CxT** | **3 DAP** | 0.020 | 0.010 | c |
| DAP | 0.12 | 1 | 51.58 | 2.56E-10 | **CxT** | **4 DAP** | 0.020 | 0.010 | b |
| cross * DAP | 0.04 | 3 | 5.91 | 1.04E-03 | **TxC** | **3 DAP** | 0.098 | 0.014 | a |
| | | | | | **TxC** | **4 DAP** | 0.134 | 0.049 | abc |
| | | | | | **TxT** | **3 DAP** | 0.124 | 0.015 | a |
| | | | | | **TxT** | **4 DAP** | 0.092 | 0.022 | a |
| | | | | | **TxT** | **3 DAP** | 0.124 | 0.02 | abc |
| | | | | | **TxT** | **4 DAP** | 0.092 | 0.022 | a |
| | | | | | **TxG** | **3 DAP** | 0.359 | 0.014 | e |
| cross | 0.449652 | 4 | 47.7833 | 2.22E-16 | **TxG** | **4 DAP** | 0.273 | 0.011 | d |
| DAP | 0.047109 | 1 | 20.0245 | 1.70E-05 | **TxG 4-2** | **3 DAP** | 0.190 | 0.009 | c |
| cross * DAP | 0.013596 | 4 | 1.44485 | 0.22 | **TxG 4-2** | **4 DAP** | 0.140 | 0.013 | abc |
| | | | | | **GxT** | **3 DAP** | 0.109 | 0.016 | a |
| | | | | | **GxT** | **4 DAP** | 0.091 | 0.034 | abc |
| | | | | | **GxG** | **3 DAP** | 0.176 | 0.011 | bc |
| | | | | | **GxG** | **4 DAP** | 0.101 | 0.013 | a |
| | | | | | **CxC** | **3 DAP** | 0.228 | 0.013 | c |
| | | | | | **CxC** | **4 DAP** | 0.098 | 0.012 | a |
| cross | 0.35 | 3 | 52.89 | 2.22E-16 | **CxG** | **3 DAP** | 0.348 | 0.012 | d |
| DAP | 0.12 | 1 | 55.89 | 1.33E-11 | **CxG** | **4 DAP** | 0.403 | 0.009 | e |
| cross * DAP | 0.16 | 3 | 24.55 | 1.78E-12 | **GxC** | **3 DAP** | 0.167 | 0.010 | b |
| | | | | | **GxC** | **4 DAP** | 0.177 | 0.033 | abc |
| | | | | | **GxG** | **3 DAP** | 0.176 | 0.010 | b |
| | | | | | **GxG** | **4 DAP** | 0.101 | 0.012 | a |
